# In vitro and in vivo studies of GAPLINC identify it as a critical host factor involved in the regulation of influenza A virus infection

**DOI:** 10.1186/s13567-026-01783-1

**Published:** 2026-07-16

**Authors:** Lulu Wang, Haiyan Xu, Jiajie Li, Qianxi Zhang, Guanghui Chui, Shihong Yan, Song Wang, Shile Huang, Ji-Long Chen

**Affiliations:** 1 Key Laboratory of Animal Pathogen Infection and Immunology of Fujian Province, College of Animal Sciences, Fujian Agriculture and Forestry University, Fuzhou, 350002 China; 2 Key Laboratory of Fujian-Taiwan Animal Pathogen Biology, College of Animal Sciences, Fujian Agriculture and Forestry University, Fuzhou, 350002 China; 3 Joint Laboratory of Animal Pathogen Prevention and Control of Fujian-Nepal, College of Animal Sciences, Fujian Agriculture and Forestry University, Fuzhou, 350002 China; 4 Department of Biochemistry and Molecular Biology, Louisiana State University Health Sciences Center, Shreveport, LA 71103 USA

**Keywords:** Influenza A virus, GAPLINC, LncRNA, ATG7, autophagy

## Abstract

Although previous studies have suggested a role for GAPLINC in regulating influenza A virus (IAV) infection, the functional involvement of GAPLINC in IAV infection in vitro and in vivo remains largely unknown. Here, we found that expression of lncRNA GAPLINC is significantly downregulated by infections with several strains of IAV, including PR8, WSN, H3N2, and H9N2. Interestingly, infections with several other viruses, such as pseudorabies virus (PRV), Sendai virus (SeV), and Herpes simplex virus (HSV), also result in a significant reduction in GAPLINC expression. During IAV infection, activation of NF-κB and the downstream IL-6/STAT3 signaling pathway contribute, at least in part, to the downregulation of GAPLINC expression. Knockdown of GAPLINC in host cells impairs the viral replication, whereas overexpression of GAPLINC increases the viral titers. Both heterozygous GAPLINC knockout (KO) mice (GAPLINC^+/-^) and homozygous GAPLINC KO mice (GAPLINC^⁻/⁻^) were further employed to determine its function in vivo. GAPLINC knockout renders mice more resistant to IAV infection than wild-type counterparts, as evidenced by lower viral load and lung injury, slower body weight loss, and improved survival. We confirmed that GAPLINC obviously suppresses the IRF3 activation in IAV-infected cells. Moreover, we noticed that inhibition of ATG7-involved autophagy weakens the pro-viral activity of GAPLINC. Together, the results support the conclusion that GAPLINC plays a critical role in enhancing the pathogenesis of IAV, at least by targeting the IRF3 and ATG7-mediated autophagy pathway.

**Supplementary Information:**

The online version contains supplementary material available at 10.1186/s13567-026-01783-1.

## Introduction

Influenza A virus (IAV), a member of the *Orthomyxoviridae* family, causes acute respiratory diseases in humans and diverse animal species, posing a significant global health threat [1]. As a single-stranded negative-sense RNA virus, IAV replication efficiency depends not only on its polymerase complex (PB1, PB2, PA), but also on complex regulation by host factors, including non-coding RNAs [2]. Recent studies reveal that long non-coding RNAs (lncRNAs) participate in viral replication and antiviral immune responses through epigenetic regulation of various genes and intricate crosstalk with autophagy processes, collectively governing outcomes of viral infection [3]. The dynamic interplay among viral exploitation of host factors, initiation of host defense mechanisms, and viral counter-hijacking of cellular pathways constitutes the core paradigm of influenza infection, thereby providing a conceptual framework for developing novel antiviral interventions [4, 5].

LncRNAs are typically defined as RNA molecules exceeding 200 nucleotides in length that lack protein-coding capacity. Although lncRNAs predominantly modulate physiological processes through multiple regulatory mechanisms, including transcriptional and post-transcriptional levels [6], dysregulation of lncRNAs is associated with numerous diseases. Emerging evidence shows that lncRNAs can encode functional micropeptides in a context-dependent manner, exerting important regulatory roles in both physiological processes and oncogenic transformation [3, 7]. During influenza virus infection, the expression of thousands of lncRNAs is significantly altered in host cells [8]. Recent studies have revealed their dual roles in IAV infection: bolstering host immune responses or being exploited by the virus to facilitate its replication. For example, IAV infection induces the expression of some lncRNAs, such as AVAN [9], IFITM4P [10], and lnc-ISG20 [11]. These lncRNAs inhibit viral replication by modulating host innate immune responses or interfering with viral replication processes [12, 13], demonstrating host self-defense mechanisms involving them. Conversely, IAV can exploit specific lncRNAs to facilitate its replication. It has been shown that lncRNA MxA suppresses IFN-β transcription by forming RNA-DNA triplexes [14]; lncRNA NSPL restrains TRIM25-mediated RIG-I ubiquitination, dampening innate immunity [15]; and lncRNA TSPOAP1*-*AS1 impairs ISRE activation and ISGs expression to promote viral replication [16]. However, the involvement of lncRNAs in the virus-host interaction and the precise mechanisms underlying the regulation of viral pathogenesis by lncRNAs remain largely unknown.

Autophagy, a highly conserved intracellular degradation and recycling mechanism, involves the formation of double-membrane vesicles (autophagosomes) that engulf damaged organelles, misfolded proteins, or invading pathogens. These cargoes are subsequently delivered to lysosomes or vacuoles for degradation. This process plays a pivotal role in maintaining cellular homeostasis, counteracting cellular stress, and regulating cell survival [17–19]. As a highly conserved degradative pathway, autophagy plays a complex yet pivotal role in host antiviral defense. Recent studies demonstrate that autophagy exerts dual effects on viral replication. On the one hand, the autophagic machinery facilitates the clearance of viral particles and components, thereby restricting viral propagation [20–22]. On the other hand, certain viruses hijack autophagic mechanisms to facilitate their own replication and dissemination. For instance, some viruses hijack autophagy-related proteins or modulate autophagic pathways to create favorable conditions for their replication [23, 24]. This dual functionality renders autophagy a multifunctional and intricate biological process implicated in virus-host interaction. IAV infection can trigger autophagosome accumulation in host cells, and autophagy plays a critical role in IAV pathogenesis [25]. IAV proteins, including M2, NP, HA, PB1, and NS1, are involved in modulating autophagic processes. Both M2 and NP proteins induce autophagy via the AKT*-*mTOR pathway [26]. Cleavage products of HA proteins from H5 and H7 subtypes upregulate LC3-II levels, suggesting an HA-mediated autophagic activation [27]. Additionally, PB1 interacts with the selective autophagy NBR1 (receptor neighbor of BRCA1 gene 1), thereby suppressing RIG-I-MAVS-mediated innate immune signaling and promoting viral infection [28]. Nevertheless, the role and underlying mechanism of autophagy in IAV infection remain incompletely defined.

Autophagy-related gene 7 (ATG7) is an essential autophagy effector enzyme. Our previous research has shown that ATG7 exerts a pivotal regulatory role in IAV-host interaction [29]. However, we found that ATG7 promoted IAV replication in autophagy-dependent and -independent manners, as inhibition of autophagy failed to completely block the upregulation of IAV replication by ATG7 [29]. To further dissect the molecular mechanisms by which ATG7 governs the influenza virus life cycle, transcriptomic profiling via RNA-seq was performed on ATG7 knockout cells infected with or without IAV. The differentially expressed lncRNAs were comprehensively analyzed to investigate their regulatory functions in IAV infection. Finally, we identified the lncRNA GAPLINC (Gastric adenocarcinoma predictive long intergenic non-coding RNA), whose known homologous sequences are conserved only in humans, certain primates, and mice, as a critical regulator involved in the promotion of IAV replication by ATG7 (Additional file 1A). Bioinformatic analysis revealed that GAPLINC has two annotated transcripts in the NCBI database and fourteen transcripts in the Ensembl genome database (Additional file 1B, C). Further analysis revealed that under IAV (PR8 strain) infection, the expression level of the human GAPLINC transcript NR_110428 (NCBI) is positively correlated with ATG7 [29].

GAPLINC, a long intergenic non-coding RNA, is significantly upregulated in multiple malignancies, including gastric, lung, and colorectal cancers. Its expression levels are strongly correlated with tumor stages and prognosis. [30]. Studies reveal that GAPLINC modulates tumor cell proliferation, invasion, and metastasis through multiple mechanisms, including ceRNA networks, signaling pathway regulation, and epigenetic modifications [31]. GAPLINC also critically regulates inflammatory responses. Notably, it acts as a negative modulator in macrophages, where its deficiency upregulates basal inflammatory genes and enhances resistance to endotoxic shock in septic mouse models [32]. However, functional involvement of GAPLINC in regulating IAV infection and antiviral immune responses remains poorly understood.

In this study, we performed both in vitro and in vivo experiments to examine the GAPLINC expression during IAV infection and to evaluate its role in the viral replication. Furthermore, we investigated the potential mechanisms underlying the function of GAPLINC in these processes, providing insights into the pathways through which GAPLINC may contribute to promotion of IAV pathogenesis.

## Materials and methods

### Cell lines and cell culture

A549 (human type II alveolar epithelial cells; ATCC^®^ CCL-185™), HEK293T (human embryonic kidney cells; ATCC^®^ CRL-3216™), MDCK (Madin-Darby canine kidney cells; ATCC^®^ CCL-34™), and HeLa (human cervical adenocarcinoma cells; ATCC^®^ CCL-2™) were purchased from American Type Culture Collection (ATCC, Manassas, VA, USA). Cells were cultured in Dulbecco's Modified Eagle Medium (DMEM) supplemented with 10% fetal bovine serum (FBS; Gibco, Waltham, MA, USA; 10437-028), penicillin (100 U/mL; Sigma-Aldrich, St. Louis, MO, USA; P7794), and streptomycin (100 μg/mL; Sigma-Aldrich; S9137). Cultures were maintained at 37 °C in a humidified 5% CO₂ atmosphere. Cells were passaged at 80–90% confluence using 0.25% trypsin–EDTA solution (Gibco; 25200072).

### Viruses and viral infection

The viruses used in this study, along with their sources where applicable, are as follows: The A/Puerto Rico/8/34 (H1N1, PR8) strain was rescued by reverse genetics using the pHW2000-based eight-plasmid system as previously described [33]. The A/WSN/33 (H1N1) influenza virus strain and the Sendai virus (SeV) Cantell strain were kindly provided by Professor George F. Gao’s laboratory at the Chinese Academy of Sciences. The H3N2 swine influenza virus (SIV) strain was derived from standard isolates maintained in our laboratory. Additionally, the H9N2 avian influenza virus strain A/Chicken/Fujian/MQ01/2015, a laboratory isolate whose full genome sequence is available in GenBank (accession numbers MT774533 to MT774540), was included [34]. Pseudorabies virus (PRV, a porcine alphaherpesvirus) and herpes simplex virus type 1 (HSV-1) were also employed. All influenza viruses and SeV were propagated in specific-pathogen-free (SPF) chicken embryos, while PRV and HSV-1 were propagated in PK-15 and Vero cells, respectively. For infection experiments, A549 cells were infected with the aforementioned viruses at the indicated multiplicity of infection (MOI). To assess the cell type specificity of IAV-induced GAPLINC downregulation, HEK293T and HeLa cells were also infected with the WSN strain. Unless otherwise specified, cells were incubated with the virus at 37 °C for 1 h, after which the inoculum was replaced with fresh maintenance medium for further culture. Since GAPLINC is a mammalian lncRNA with no known homolog in avian species, all functional analyses were performed in mammalian cells. Viruses from different host origins were included to assess whether the regulatory effects of GAPLINC on viral infection are conserved across multiple IAV subtypes.

### Generation of stable cell lines

Briefly, stable A549 cell lines were generated using lentiviral expression systems. For knockdown, A549 cells were infected with lentiviruses expressing specific shRNAs from the pSIH-H1-GFP vector. For overexpression, the full-length human GAPLINC sequence (NCBI Accession: NR_110428.1) was cloned into the pNL lentiviral vector. To generate A549 cells expressing constitutively active STAT3, the pLVX3-STAT3^D661V^ plasmid containing constitutively active form of STAT3 was constructed as described previously [35]. Lentiviral particles carrying either pLVX3-STAT3^D661V^ or the pLVX3 empty vector were generated and used to transduce A549 cells. After transduction, cells were selected with puromycin to establish stable A549 cell lines expressing constitutively active STAT3 or the corresponding empty vector control. The shRNA sequences are listed in Table 1.

**Table 1:** **Sequences of shRNAs used in this study**

shRNAs	Sequences (5’−3’)
sh-GAPLINC	GCCAATGCCTGAAATAATGAA
sh-ATG7	GCTCTTCCTTACTTCTTAATC
sh-STAT3	GCAGCAGCTGAACAACATG

### Nuclear and cytoplasmic fractionation

Nuclear and cytoplasmic fractions were prepared from A549 cells using the PARIS™ Kit (Thermo Fisher Scientific, Waltham, MA, USA; Cat. No. AM1921) according to the manufacturer’s instructions. RNA was isolated independently from the nuclear and cytoplasmic fractions and used for qRT-PCR and RT-PCR analyses. U6 small nuclear RNA (snRNA) was used as a nuclear marker to assess the fractionation efficiency.

### Viral attachment assay

A549 cells were seeded uniformly in 6-well plates and cultured until reaching approximately 90% confluence. Before the experiment, the culture plates were pre-chilled at 4 °C for 10 min. The cells were then washed twice with ice-cold phosphate buffered saline (PBS) to thoroughly remove residual medium. Subsequently, diluted PR8 influenza virus solution was added at the indicated MOIs, and the plates were incubated at 4 °C for 1 h. After adsorption, the cells were washed five times with ice-cold PBS to remove unbound viral particles. Finally, cells were lysed to extract viral genomic RNA, and the cell-associated viral RNA levels were quantified by qRT-PCR to evaluate attachment efficiency.

### Viral internalization assay

Viral internalization was assessed following the attachment assay. First, synchronized viral attachment was performed as described in the Viral Attachment Assay section by incubating the virus with cells at 4 °C for 1 h. After adsorption, unbound viruses were removed by washing three times with ice-cold PBS. Then, 2 mL of pre-warmed virus maintenance medium was added, and the plates were transferred to a 37 °C, 5% CO₂ incubator and incubated for 30 min to permit viral internalization. After incubation, cells were treated three times with acidic Glycine–HCl buffer (pH = 1.3) to degrade any non-internalized virus particles remaining on the cell surface, thereby ensuring that only internalized viruses were detected. Cells were then lysed, and internalized viral vRNA was quantified by qRT-PCR.

### RNA preparation, RT-PCR, and quantitative real-time PCR

Total RNA was extracted using TRIzol reagent (TIANGEN, Beijing, China; ZK201) according to the manufacturer’s instructions. cDNA was synthesized using the HiScript III 1 st Strand cDNA Synthesis Kit (Vazyme, Nanjing, China; R312-01). The resulting cDNA was subjected to PCR using Taq DNA polymerase (GenStar Biotechnology, Beijing, China; ZA002-101S) or to quantitative real-time PCR using SYBR Green Master Mix (Vazyme; Q321-02). The sequences of all primers used in this study are listed in Table 2. The primers designated as “18S rRNA” were used to detect 18S rRNA as an internal reference for the analysis of IAV vRNA and cRNA as described previously [36]. For expression analysis of host genes by PCR and qRT-PCR, GAPDH was used as the reference gene in human cell experiments and β-actin was used in mouse samples. In western blotting analyses, β-actin was used as the loading control in both human and mouse samples.

**Table 2:** **Sequences of primers used in this study**

Gene Name	Sequences
GAPLINC (human)	Forward: TGACACATCCTCTTGGTTTCCT Reverse: TCTGTGCATACCCTGAGTCC
GAPLINC (mouse)	Forward: GGGCTCTAGAGTTCCGTTCTC Reverse: CCCCAAGTCGACTTCCTACTGA
GAPDH (human)	Forward: TGGGTGTGAACCATGAGAAGT Reverse: AAGGCCATGCCAGTGAGCTT
NP	Forward: TCAAACGTGGGATCAATG Reverse: GTGCAGACCGTGCTAGAA
PRV-gE	Forward: CTTCCACTCGCAGCTCTTCT Reverse: TAGATGCAGGGCTCGTACAC
H9N2-NP	Forward: CAACCATTATGGCAGCATT Reverse: TACTCCTCTGCATTGTCTCC
SeV-NP	Forward: ATAAGTCGGGAGGAGGTGCT Reverse: GTTGACCCTGGAAGAGTGGG
β-actin (mouse)	Forward: AATGGGTCAGAAGGACTCCT Reverse: ACGGTTGGCCTTAGGGTTCAG
IL-6 (human)	Forward: AATGAGGAGACTTGCCTGGTG Reverse: TGAGGTGCCCATGCTACATT
18S rRNA	Forward: GTAACCCGTTGAACCCCATT Reverse: CCATCCAATCGGTAGTAGCG
vRNA for RT	AGCGAAAGCAGG
cRNA for RT	AGTAGAAACAAGG

### Antibodies and reagents

Following antibodies were used in this study: anti-ATG7 (Proteintech, Wuhan, China; 67341−1-Ig); anti-p65 (Cell Signaling Technology, Danvers, MA, USA; 8242), anti-phospho-p65 (Ser536) (Cell Signaling Technology; 3033), anti-STAT3 (Cell Signaling Technology; 9139), and anti-phospho-STAT3 (Tyr705) (Cell Signaling Technology; 9131); anti-LC3 (Sigma-Aldrich; L7543); and anti-β-actin (TransGen Biotech, Beijing, China; HC-201-02). Following reagents were used in this study: dimethyl sulfoxide (DMSO) (Sigma-Aldrich; D8371); Tocilizumab (MedChemExpress, Monmouth Junction, NJ, USA; HY-P9917), chloroquine (CQ) (MedChemExpress; 6628-25G); lipopolysaccharide (LPS) (Sigma-Aldrich; L4391); and interleukin-6 (IL-6) (PeproTech, Cranbury, NJ, USA; 200-06).

### Western blotting

Cells were lysed with radioimmunoprecipitation assay (RIPA) buffer supplemented with protease and phosphatase inhibitors. Immunoprecipitates were washed three times with lysis buffer, separated by SDS-PAGE, transferred onto a nitrocellulose membrane, and probed with antibodies as indicated. Semi-quantification of blots was performed using ImageJ software.

### Cell death detection

Cell death was evaluated by propidium iodide (PI) staining and fluorescence microscopy. Briefly, A549 cells were infected with the influenza PR8 virus at an MOI of 1 for 36 h. Afterwards, the cells were stained with PI, and PI-positive (red fluorescence) cells were defined as dead. Representative fluorescence images were captured. For quantification, at least three random fields per condition were imaged under both bright-field and fluorescence (PI) channels. Total cell counts were determined from bright-field images, while PI-positive cells were identified from the PI channel using a set threshold in ImageJ. The cell death rate was calculated as (PI-positive cells/total cells) × 100%. Experiments were performed in triplicate.

### Animal experiments

This study utilized wild-type (WT), GAPLINC heterozygous (GAPLINC^+/-^), and homozygous knockout (GAPLINC^⁻/⁻^) mice on a C57BL/6 J background (aged 5–6 weeks) to investigate the role of GAPLINC in viral infection. For viral infection, mice were anesthetized via intraperitoneal injection of Zoletil-50 (50 mg/kg) and inoculated intranasally with 50 µL of each virus including PR8, WSN, H9N2 and PRV at indicated doses, or mock treatment. The viral doses used in the experiments are shown in corresponding figure legends. Survival and body weight were recorded daily for 14 days. For endpoint analyses, a separate cohort of mice was euthanized at 48 h post-infection. Lung viral load was quantified by plaque assay, and viral NP gene expression was measured by qRT-PCR. Histopathological assessment of lung injury was performed by an evaluator blinded to group allocation. In survival studies, group sizes for survival studies were n = 8 (infected) and n = 3 (mock) per genotype; for terminal analysis, *n* = 5 (WSN) or *n* = 3 (other viruses) per genotype, with corresponding controls.

### Statistical analysis and reproducibility

All data were analyzed using GraphPad Prism software (version 8.0.1) and are presented as the mean ± standard deviation (SD). For in vitro experiments, statistical significance was determined by Student’s t-test, and each experiment was independently repeated at least three times. For in vivo experiments, data from each genotype (WT, GAPLINC^+/-^, GAPLINC^⁻/⁻^) were analyzed and are presented independently; statistical comparisons between virus-infected groups and their genotype-matched controls under the same condition were performed using t-tests. *P* < 0.05 was considered statistically significant.

## Results

### Viral infections downregulate the expression of GAPLINC in vitro and in vivo

**Figure 1:**
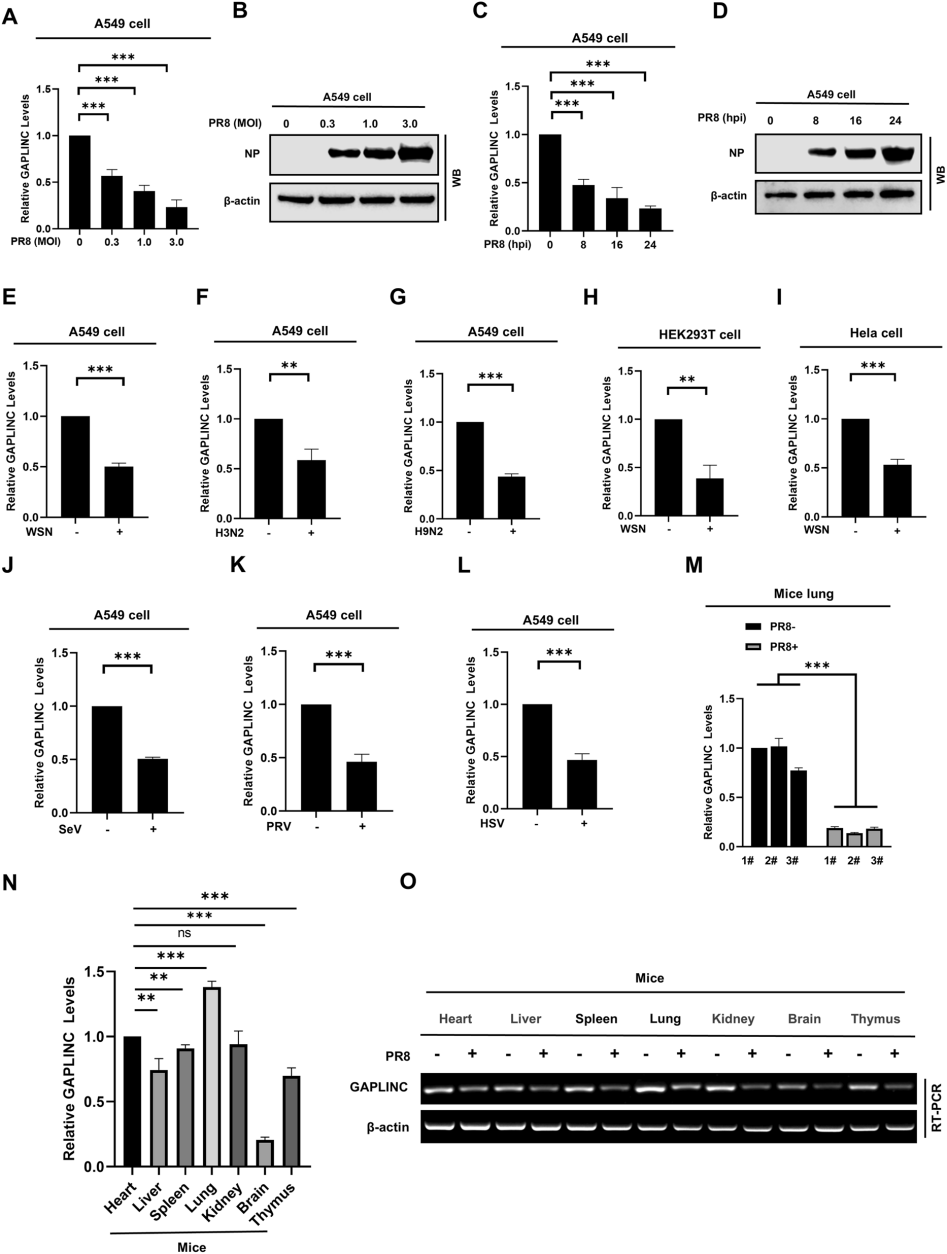
**Viral infections downregulate the expression of GAPLINC in vitro and in vivo***.* **A**, **B** qRT-PCR analysis of GAPLINC RNA levels in A549 cells infected with PR8 at an MOI of 0, 0.3, 1, or 3 and harvested at 16 hpi (**A**, n = 3). Viral NP protein was detected by western blotting (**B**). **C**, **D** qRT-PCR analysis of GAPLINC RNA levels in A549 cells infected with PR8 at an MOI of 1 and harvested at 0, 8, 16, or 24 hpi (**C**, n = 3). Viral NP protein was detected by western blotting (**D**). **E**–**G** qRT-PCR analysis of GAPLINC RNA levels in A549 cells at 16 hpi with WSN (MOI = 1) (**E**, n = 3), H3N2 (MOI = 1) (**F**, n = 3), or H9N2 (MOI = 1) (**G**, n = 3). **H-I** qRT-PCR analysis of GAPLINC RNA levels in HEK293T cells (**H**, n = 3), HeLa cells (**I**, n = 3) at 16 hpi with WSN (MOI = 1). **J**–**L** qRT-PCR analysis of GAPLINC RNA levels in A549 cells at 16 hpi with SeV (MOI = 1) (**J**, n = 3), PRV (MOI = 1) (**K**, n = 3) and HSV (MOI = 1) (**L**, n = 3). **M** qRT-PCR analysis of GAPLINC RNA levels in the indicated tissues of WT mice (5–6 weeks) at 48 h post intranasal infection with PR8 (2 × 10^3^ PFU, n = 3/group). **N** qRT-PCR analysis showing the basal expression levels of GAPLINC in the indicated tissues from uninfected mice (5–6 weeks, n = 3). **O** Representative RT-PCR images showing GAPLINC levels in the indicated tissues of WT mice (5–6 weeks), either non-infected (mock) or infection with PR8 for 48 h. Representative data from three biologically independent experiments are shown. Data are presented as means ± SD, not significant (ns), **P < 0.01 and ***P < 0.001.

To investigate whether infections with different viruses affect GAPLINC expression, several viruses were used. We found that infection with different MOI of IAV strain PR8 caused a significant decrease in expression of GAPLINC in A549 cells (Figure 1A, B, Additional file 2A). Similar results were obtained from a time course study with the PR8 virus (Figure 1C, D, Additional file 2B). Further experiments demonstrated that A549 cells infected with multiple other IAV strains including WSN, H3N2 and H9N2 also resulted in a reduction of GAPLINC expression (Figure 1E–G; Additional file 2C-H). Consistently, IAV strain WSN infection of HEK293T and HeLa cells significantly downregulated the GAPLINC expression (Figures 1H, I, Additional file 2I, J). Moreover, following infection of A549 cells with other RNA viruses (Sendai virus, SeV) or DNA viruses, including Pseudorabies virus (PRV) and Herpes Simplex virus (HSV), the expression of GAPLINC was similarly reduced (Figure 1J–L; Additional file 2 K–O), suggesting that downregulation of GAPLINC by viral infection may represent a broad-spectrum host response.

In vivo experiments using a mouse model further revealed that IAV PR8 infection significantly downregulated GAPLINC expression in the animals' lung tissues (Figure 1M; Additional file 2P, Q). We also examined the basal expression of GAPLINC in different tissues from uninfected mice and found that its expression varied among organs (Figure 1N). Expression of GAPLINC and viral NP was then assessed in different tissues at 48 h post PR8 infection (Figure 1O, Additional file 2R).

### IL-6/STAT3 signaling regulates the expression of GAPLINC

**Figure 2:**
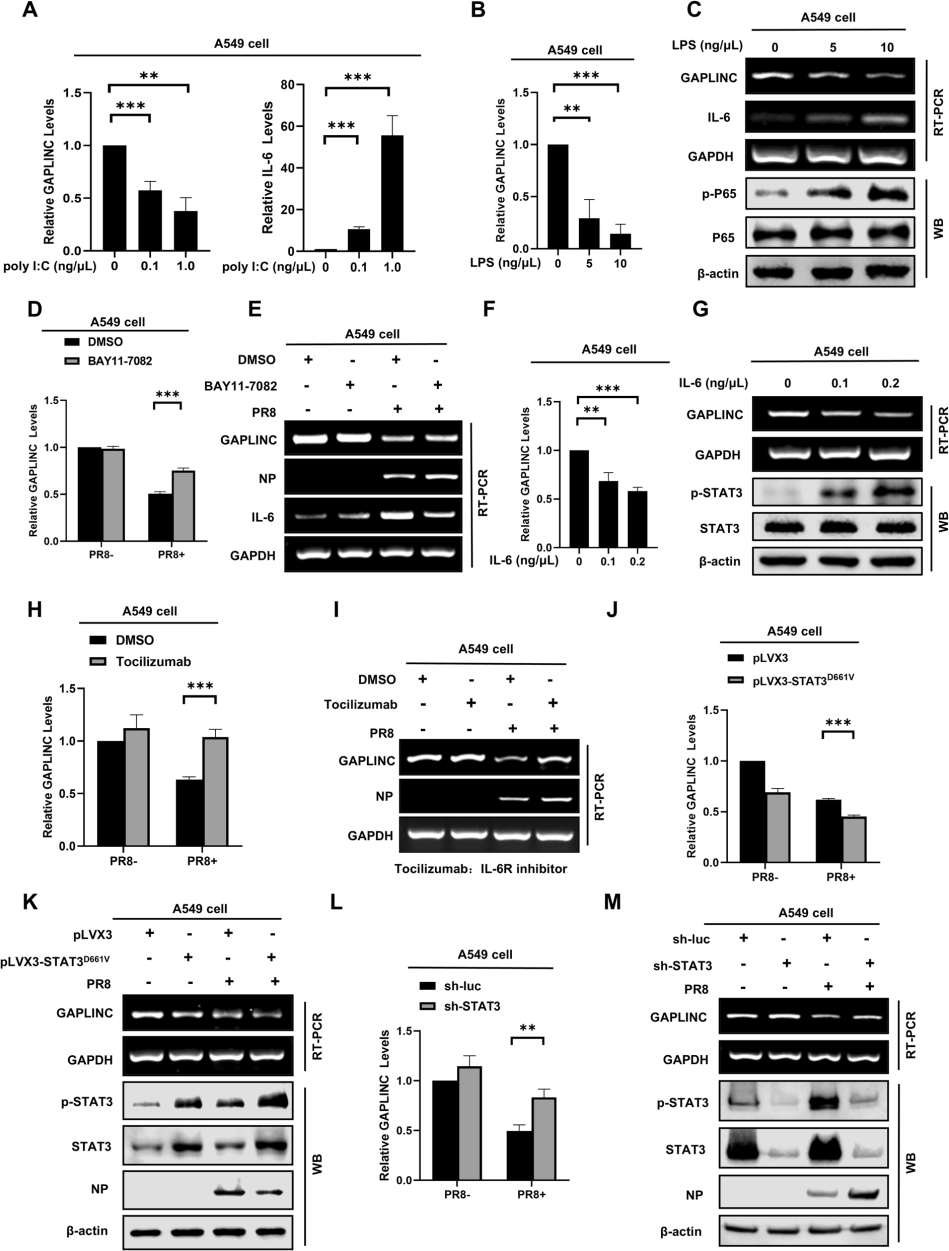
**IL-6/STAT3 signaling regulates the expression of GAPLINC.** **A** qRT-PCR analysis of GAPLINC and IL-6 RNA levels in A549 cells stimulated with poly I:C (0, 0.1, or 1.0 ng/μL) for 12 h (n = 3). **B**, **C** qRT-PCR (**B**, n = 3) analysis and RT-PCR combined with western blotting (**C**) analysis of GAPLINC expression in A549 cells treated with LPS (0, 5, or 10 ng/μL) for 6 h. **D**, **E** GAPLINC RNA levels detected by qRT-PCR (**D**, n = 3) and RT-PCR (**E**) in A549 cells pretreated with BAY11-7082 (20 nM) or DMSO for 1 h, followed by PR8 infection (MOI = 1, 16 hpi). **F**, **G** qRT-PCR (**F**, n = 3) and RT-PCR (**G**) analysis of GAPLINC RNA levels in A549 cells treated with IL-6 (0, 0.1, or 0.2 ng/μL) for 6 h. **H**, **I** qRT-PCR (**H**, n = 3) and RT-PCR (**I**) analysis of GAPLINC in A549 cells pretreated with tocilizumab (IL-6R inhibitor) or DMSO for 2 h, followed by PR8 infection (MOI = 1, 16 hpi). **J**, **K** qRT-PCR (**J**, n = 3) and RT-PCR (**K**) analysis of GAPLINC in A549 cells stably expressing constitutively active STAT3 (pLVX3-STAT3^D661V^) or the corresponding vector control (pLVX3) following PR8 infection (MOI = 1, 8 hpi). **L**, **M** qRT-PCR (**L**, n = 3) and RT-PCR (**M**) analysis of GAPLINC in STAT3-knockdown A549 cells or the corresponding control cells following PR8 infection (MOI = 1, 8 hpi). **K** and **M** represent two distinct A549 cell models showing effects of STAT3 overexpression and knockdown on GAPLINC expression. Shown are representative data from three biologically independent experiments. Data are presented as means ± SD, **P < 0.01 and ***P < 0.001.

Having observed the downregulation of GAPLINC expression induced by viral infection, we next determined whether innate immune signaling regulates GAPLINC expression, as it is activated during viral infection. To address this possibility, A549 cells were treated with poly I:C, a synthetic analog of viral double-stranded RNA that activates multiple pattern recognition receptors (PRRs) and PRR-dependent immune signaling. The experiments demonstrated that poly I:C treatment led to the downregulation of GAPLINC expression (Figure 2A), suggesting the involvement of innate immune signaling in regulating this process. Based on our previous finding that the NF-κB pathway mediates expression of GAPLINC [29], we further examined the relationship between the dynamics of GAPLINC expression and activation of the NF-κB pathway during IAV infection. For this, A549 cells were treated with LPS, a known activator of NF-κB signaling. The results showed that LPS treatment markedly downregulated GAPLINC expression (Figure 2B, C), suggesting that the viral infection-mediated downregulation of GAPLINC might be associated with the NF-κB-dependent pathway. To verify this, cells were pretreated with the NF-κB inhibitor BAY11-7082, followed by infection with the PR8 virus. The results revealed that inhibition of NF-κB significantly attenuated the downregulation of GAPLINC by the viral infection (Figure 2D, E). Collectively, the above observations indicate that downregulation of GAPLINC by viral infection is likely regulated by NF-κB-dependent innate immune signaling.

To confirm this finding, IL-6 was used since it is a key cytokine whose expression is governed by NF-κB. Interestingly, IL-6 stimulation was found to effectively downregulate the GAPLINC expression in A549 cells (Figure 2F, G). Conversely, inhibition of IL-6 receptor with tocilizumab increased GAPLINC expression (Figure 2H, I). Given that STAT3 is a downstream effector of NF-κB/IL-6 signaling, we next examined whether STAT3 is involved in the regulation of GAPLINC expression during viral infection. As expected, in A549 cells stably expressing STAT3^D661V^, a constitutively active form of STAT3, GAPLINC levels were significantly reduced both in the presence and absence of PR8 infection (Figure 2J, K). Conversely, in STAT3-knockdown A549 cells, GAPLINC expression was increased, and the virus-induced downregulation of GAPLINC was attenuated (Figure 2L, M). Together, these results suggest that the IL-6/STAT3 pathway negatively regulates GAPLINC expression during IAV infection.

### Knockdown of GAPLINC suppresses influenza virus replication

**Figure 3:**
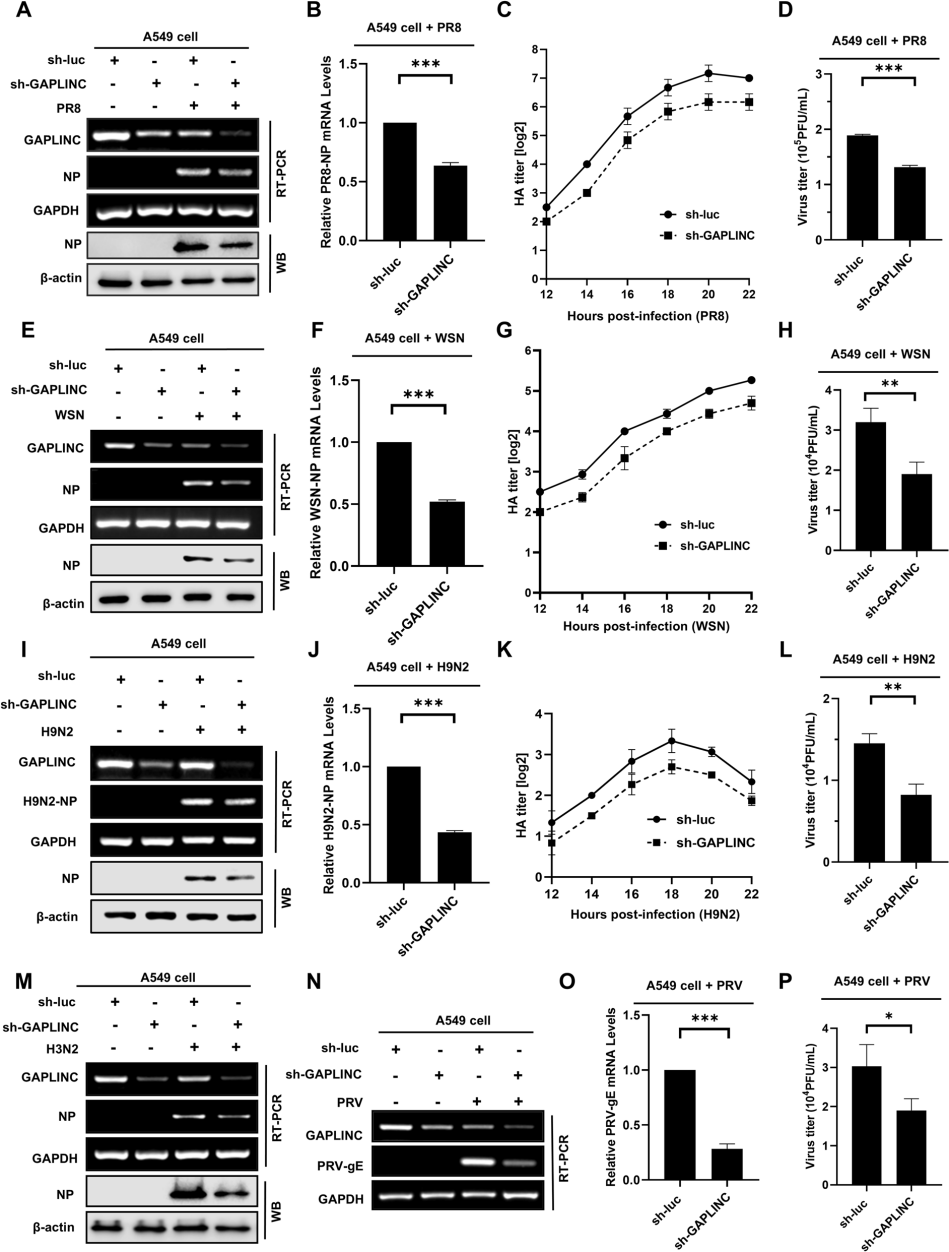
**Knockdown of GAPLINC suppresses influenza virus replication.** **A**–**H** Analysis of PR8 (MOI = 1) (**A**–**D**) or WSN (MOI = 1) infection (**E**–**H**) in GAPLINC-knockdown and control A549 cell lines 16 hpi. Viral NP mRNA and protein expression in cell lysates detected by RT-PCR and western blotting (**A**, **E**). Viral NP mRNA levels quantified by qRT-PCR (**B**, **F**, n = 3). Hemagglutination assay (HA) (**C**, **G**, n = 3) and plaque assay (**D**, **H**, n = 3) of viral titers in the cell culture supernatants. **I**–**L** Analysis of H9N2 (MOI = 1) infection in GAPLINC-knockdown and control A549 cell lines 16 hpi. Viral NP mRNA and protein expression in cell lysates detected by RT-PCR and western blotting (**I**). Viral NP mRNA levels quantified by qRT-PCR (**J**, n = 3). Hemagglutination assay (HA) (**K**, n = 3) and plaque assay (**L**, n = 3) of viral titers in the cell culture supernatants. **M** Analysis of H3N2 (MOI = 1) infection in GAPLINC-knockdown and control A549 cell lines 16 hpi. Viral NP mRNA and protein expression in cell lysates detected by RT-PCR and western blotting. **N**–**P** Analysis of pseudorabies virus (PRV, MOI = 1) infection in GAPLINC-knockdown and control A549 cell lines 16 hpi. Viral PRV-gE mRNA levels detected by RT-PCR (**N**). Viral PRV-gE mRNA levels quantified by qRT-PCR (**O**, n = 3). Plaque assay of viral titers in the cell culture supernatants (**P**, n = 3). Shown are representative data from three biologically independent experiments. Data are presented as means ± SD, *P < 0.05, **P < 0.01 and ***P < 0.001.

To elucidate the role of GAPLINC in influenza virus infection, stable GAPLINC-knockdown A549 cell lines were generated and then challenged with IAV strain PR8. The cytopathic effect (CPE) experiments demonstrated that GAPLINC-knockdown cells were more resistant to virus-induced cellular pathological damage than the control cells (Additional file 3A). To quantitatively assess the extent of cell death, we performed propidium iodide (PI) staining. Consistent with the CPE data, knockdown of GAPLINC significantly reduced the proportion of PI-positive cells compared to the control (Additional file 3B, C). In addition, virus-infected and non-infected samples of total RNA and proteins were harvested and examined by RT-PCR, qRT-PCR, and western blotting. Viral titers in the cell culture supernatants were assessed by hemagglutination assay (HA) and plaque formation assay (PFA). The results revealed that knockdown of GAPLINC led to a significant attenuation of IAV replication, as indicated by impaired mRNA and protein expression of viral NP, and decreased viral titers (Figure 3A–D; Additional file 3D).

In line with the above finding, similar results were obtained from the experiments using other IAV strains including WSN (Figure 3E–H), H9N2 (Figure 3I–L), and H3N2 (Figure 3M), as evidenced by the reduced viral NP and viral titers. Besides, knockdown of GAPLINC also resulted in a significant decrease in DNA virus PRV gE expression and its replication (Figure 3N–P). The results indicate that knockdown of GAPLINC can suppress the replication of both RNA virus IAV and DNA virus PRV.

### Overexpression of GAPLINC promotes viral replication

**Figure 4:**
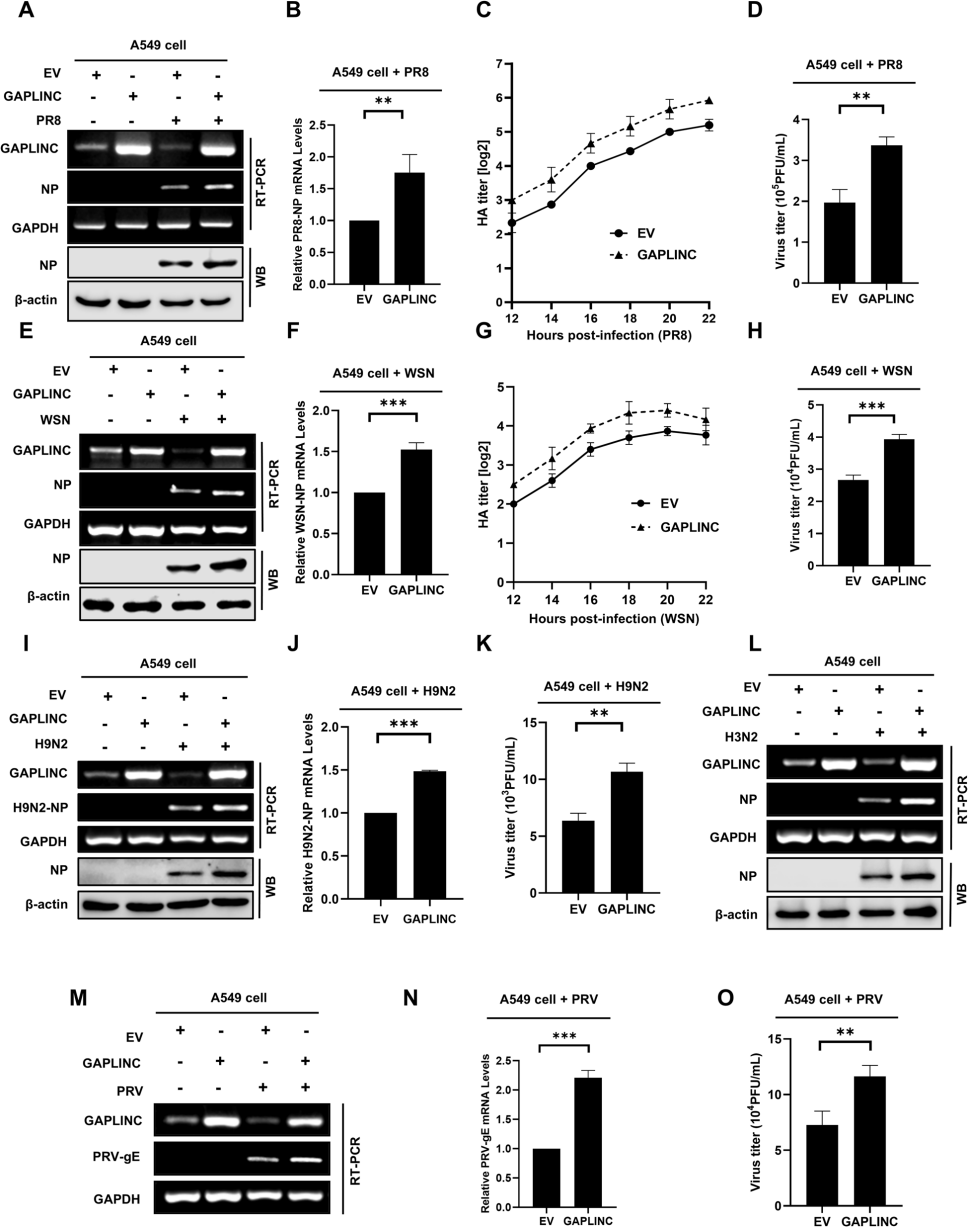
**Overexpression of GAPLINC promotes viral replication.** **A**–**H** Analysis of PR8 (MOI = 1) (**A**–**D**) or WSN (MOI = 1) infection (**E**–**H**) in GAPLINC-overexpressing and control A549 cell lines 16 hpi. Viral NP mRNA and protein expression in cell lysates detected by RT-PCR and western blotting (**A**, **E**). Viral NP mRNA levels quantified by qRT-PCR (**B**, **F**, n = 3). Hemagglutination assay (HA) (**C**, **G**, n = 3) and plaque assay (**D**, **H**, n = 3) of viral titers in the cell culture supernatants. **I**–**K** Analysis of H9N2 (MOI = 1) infection in GAPLINC-overexpressing and control A549 cell lines 16 hpi. Viral NP mRNA and protein expression in cell lysates detected by RT-PCR and western blotting (**I**). Viral NP mRNA levels quantified by qRT-PCR (**J**, n = 3). Plaque assay of viral titers in the cell culture supernatants (**K**, n = 3). **L** Analysis of H3N2 (MOI = 1) infection in GAPLINC-overexpressing and control A549 cell lines 16 hpi. Viral NP mRNA and protein expression in cell lysates detected by RT-PCR and western blotting. **M**–**O** Analysis of pseudorabies virus (PRV, MOI = 1) infection in GAPLINC-overexpressing and control A549 cell lines 16 hpi. Viral PRV-gE mRNA levels detected by RT-PCR (**M**). Viral PRV-gE mRNA levels quantified by qRT-PCR (**N**, n = 3). Plaque assay of viral titers in the cell culture supernatants (**O**, n = 3). Shown are representative data from three biologically independent experiments. Data are presented as means ± SD, **P < 0.01 and ***P < 0.001.

To corroborate the functional relevance of GAPLINC in viral replication, A549 cell lines stably overexpressing GAPLINC were further generated. As expected, overexpression of GAPLINC potently enhanced virus-mediated cytopathic damage compared to the control (Additional file 3E). In line with this, overexpression of GAPLINC resulted in a significant increase in the proportion of PI-positive cells (Additional file 3F, G). Using this model, we examined the effect of GAPLINC overexpression on the replication of the IAV strain PR8. Samples were collected synchronously at 16 hpi and analyzed by RT-PCR, qRT-PCR, western blotting, HA, and PFA. Indeed, levels of both IAV PR8 NP mRNA and protein were clearly elevated in GAPLINC-overexpressing cells compared to controls (Figure 4A, B). Consistently, overexpression of GAPLINC resulted in an increase in viral titers of PR8 (Figure 4C, D). Furthermore, similar results were obtained from experiments using other IAV strains, including WSN (Figure 4E–H), H9N2 (Figure 4I–K), H3N2 (Figure 4L), and the DNA virus PRV (Figure 4M–O). Collectively, these findings suggest a critical role for GAPLINC in the replication of diverse viral pathogens.

### GAPLINC facilitates influenza virus replication at a post-entry stage

**Figure 5:**
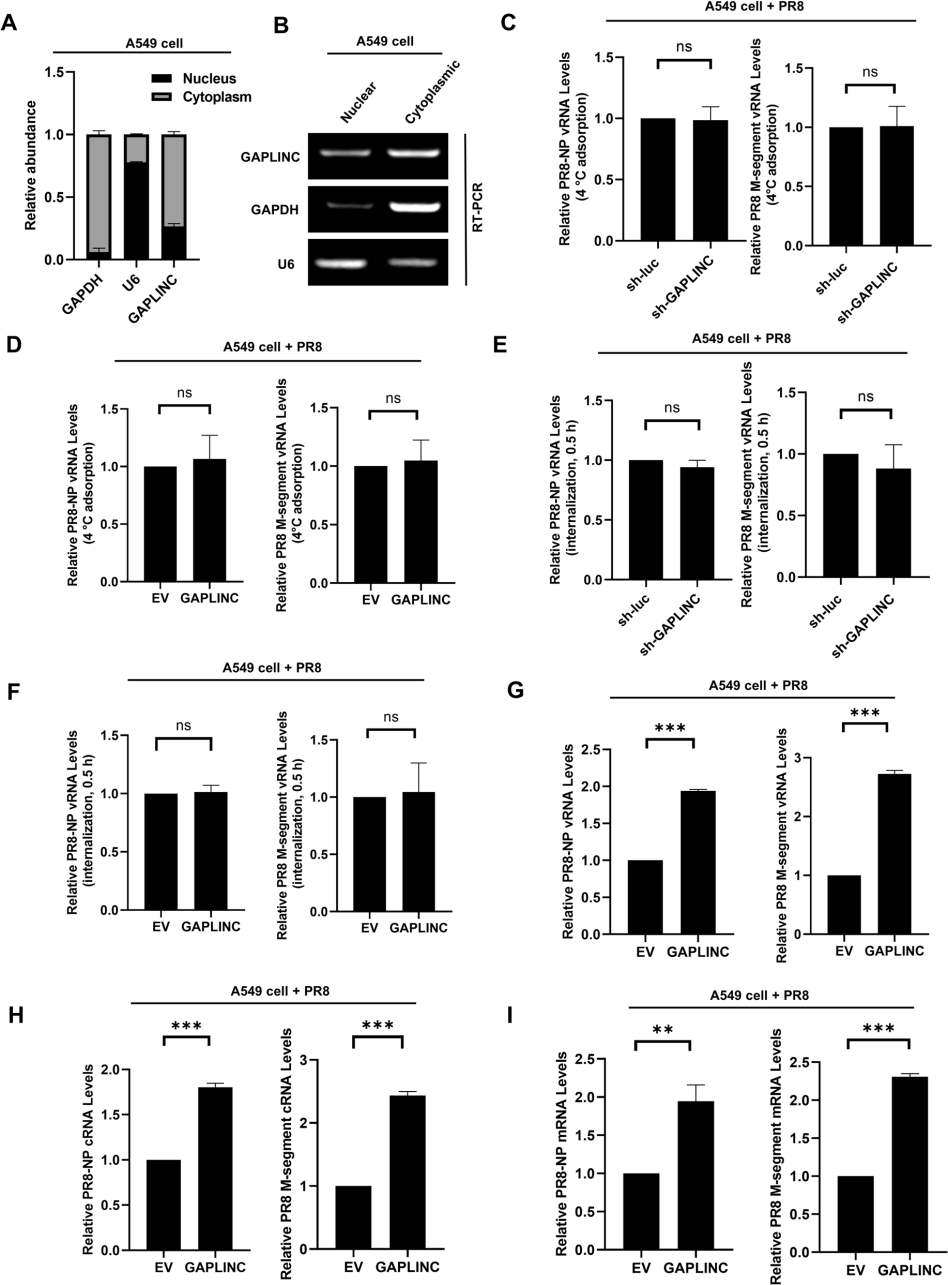
**GAPLINC facilitates influenza virus replication at a post-entry stage.** **A**, **B** Subcellular fractionation was performed and GAPLINC levels in nuclear and cytoplasmic fractions of A549 cells were detected by qRT-PCR (**A**, n = 3) and RT-PCR (**B**). U6 small nuclear RNA (snRNA) and GAPDH were used as nucleus and cytoplasm markers, respectively. **C**, **D** GAPLINC-knockdown (**C**), GAPLINC-overexpressing (**D**), and corresponding control A549 cells were incubated with PR8 virus (MOI = 5) at 4 °C for 1 h to allow synchronous viral attachment while preventing internalization. The levels of cell-associated vRNA for the viral NP and M genes were quantified by qRT-PCR (**C**, **D**, n = 3). **E**, **F** GAPLINC-knockdown (**E**), GAPLINC-overexpressing (**F**), and corresponding control A549 cells were incubated with PR8 virus (MOI = 5) attachment at 4 °C for 1 h, followed by a temperature shift to 37 °C for 30 min to allow internalization. The levels of internalized vRNA for the NP and M genes were then quantified by qRT-PCR (**E**, **F**, n = 3). **G**–**I** qRT-PCR analysis of vRNA (**G**), cRNA (**H**), and mRNA (**I**) levels for both NP and M genes in the control and GAPLINC-overexpressing A549 cells at 16 hpi with PR8 (MOI = 1) (**G**–**I**, n = 3). Shown are representative data from three biologically independent experiments. Data are presented as means ± SD, not significant (ns), **P < 0.01 and ***P < 0.001.

We next determined how GAPLINC was implicated in viral replication. To this end, the subcellular localization of GAPLINC was first assessed using qRT-PCR-based quantification of nuclear and cytoplasmic fractions. U6 small nuclear RNA (snRNA) and GAPDH were used as nucleus and cytoplasm markers, respectively. We observed that GAPLINC was localized in both the cytoplasm and nucleus, but was predominantly enriched in the cytoplasmic compartment (Figure 5A, B). Subsequently, we examined the specific stages of the influenza virus life cycle that GAPLINC may impact. Stable A549 cell lines with either GAPLINC-knockdown, GAPLINC-overexpression, or controls were used, and cell-associated vRNA levels were measured following the virus adsorption and early internalization. Quantitative analysis revealed no significant difference in vRNA levels between either GAPLINC-knockdown or -overexpressing cells and their respective controls at the early time points (1–1.5 h) (Figure 5C–F; Additional file 4A–D), suggesting that altering GAPLINC expression had no significant effect on viral attachment or internalization.

We then asked whether GAPLINC influences viral RNA synthesis in host cells. For this, the kinetics of viral replication in GAPLINC-overexpressing cells was analyzed by quantifying three viral RNA species including viral genomic RNA (vRNA), complementary RNA (cRNA), and mRNA. Strikingly, the results showed that overexpression of GAPLINC potently enhanced viral RNA synthesis compared to the control, as evidenced by a significant increase in the intracellular accumulation of all three viral RNA species in GAPLINC-overexpressing cells infected with PR8 (Figure 5G–I). Consistent with this gain-of-function phenotype, knockdown of GAPLINC exhibited an opposite effect, significantly reducing the levels of all three viral RNA species (Additional file 4E–G). Together, these data indicate that GAPLINC is a critical factor that facilitates influenza virus replication at a post-entry step, i.e., viral RNA synthesis.

### Heterozygous GAPLINC knockout mice are resistant to IAV infection

**Figure 6:**
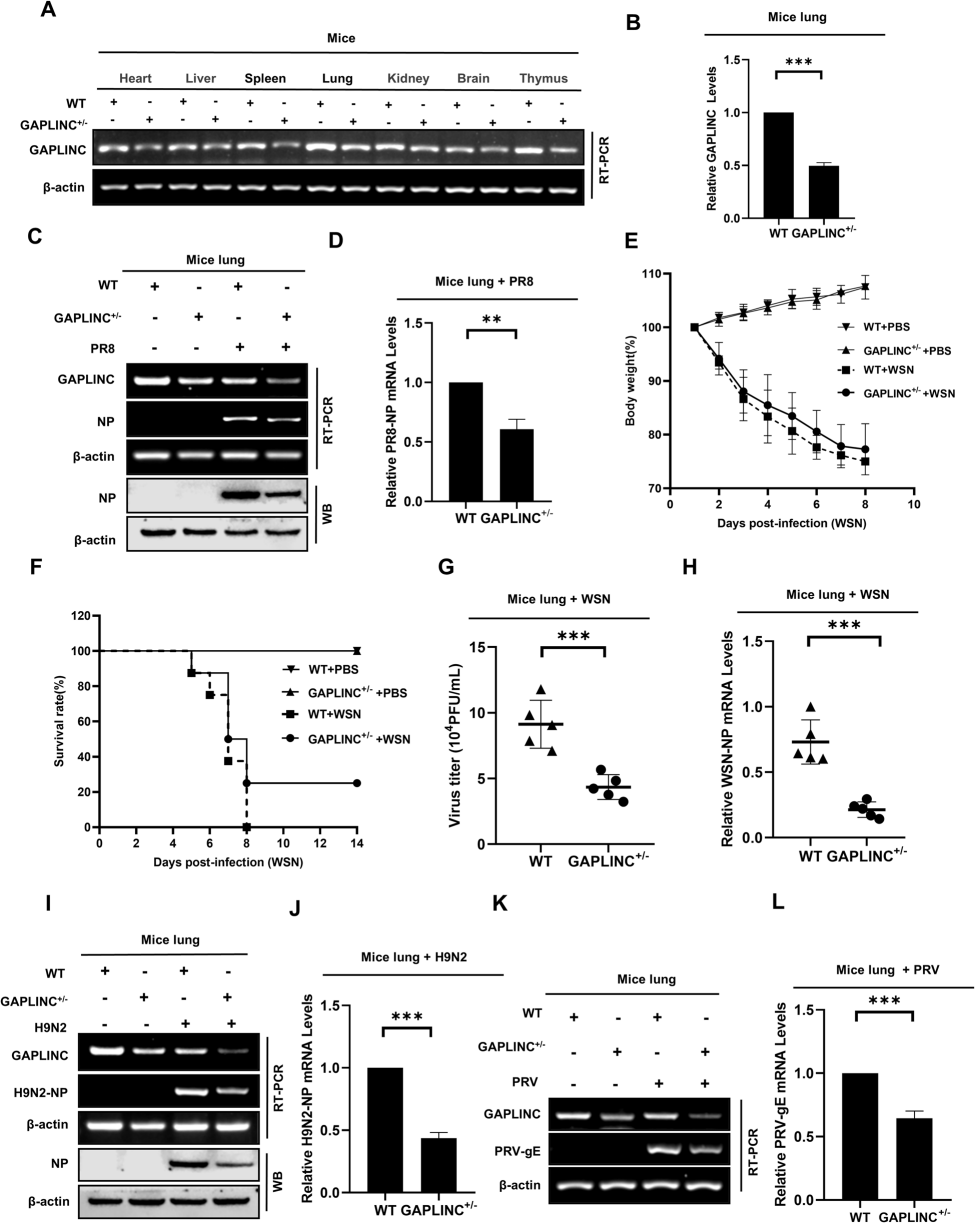
**Heterozygous GAPLINC knockout mice are resistant to IAV infection.** **A**, **B** GAPLINC RNA expression in the indicated tissues of WT and GAPLINC^+/-^ mice detected by RT-PCR (**A**) or qRT-PCR (**B**, n = 3/group). **C**, **D** Analysis of viral NP in lungs of WT and GAPLINC^+/-^ mice at 48 hpi with PR8 (2 × 10^3^ PFU). Viral NP mRNA and protein expression in cell lysates detected by RT-PCR and western blotting (**C**). Viral NP mRNA levels quantified by qRT-PCR (**D**, n = 3/group). **E**, **F** Body weight changes (**E**) and survival rates (**F**) of WT and GAPLINC^+/-^ mice (5–6 weeks, n = 8/group) following infection with the WSN virus (2 × 10^3^ PFU). **G**, **H** Viral titers measured by plaque assay (**G**) and NP mRNA levels assessed by qRT-PCR (**H**) in lungs of WSN-infected (2 × 10^3^ PFU) WT and GAPLINC^+/-^ mice (n = 5/group) at 48 hpi. **I**, **J** Analysis of viral NP in lungs of WT and GAPLINC^+/-^ mice at 48 hpi with H9N2 (1 × 10^5^ PFU). Viral NP mRNA and protein expression in cell lysates detected by RT-PCR and western blotting (**I**). Viral NP mRNA levels quantified by qRT-PCR (**J**, n = 3/group). **K, L**) Viral PRV-gE mRNA levels from WT and GAPLINC^+/-^ mice at 48 hpi PRV (1 × 10^5^ PFU) infection detected by RT-PCR (**K**) and qRT-PCR (**L**, n = 3/group). Shown are representative data from three biologically independent experiments. Data are presented as means ± SD, **P < 0.01 and ***P < 0.001.

To validate the in vitro regulatory role of GAPLINC in IAV infection of host cells, a GAPLINC-knockout murine model was utilized. To this end, heterozygous GAPLINC knockout mice (GAPLINC^+/-^) were commercially obtained from GemPharmatech (Nanjing, China). Compared with wild-type (WT) littermates, GAPLINC^+/-^ mice showed no discernible differences in body weight, body length, coat condition, or locomotor activity (Additional file 5B), indicating normal baseline physiological conditions. Our RT-PCR analyses confirmed a clear reduction in GAPLINC expression in the heart, liver, spleen, lung, kidney, brain, and thymus of GAPLINC^+/-^ mice compared to WT littermates (Figure 6A, B). Following validation of the knockout model, we initially assessed the in vivo role of GAPLINC using influenza virus PR8. Infection of GAPLINC^+/-^ mice revealed that GAPLINC deficiency significantly decreased viral NP expression in lungs compared to WT controls (Figure 6C, D). To further characterize the protective phenotype under varying virulence conditions, we employed another IAV strain, WSN. Accordingly, WSN infection showed that GAPLINC^+/-^ mice exhibited less weight loss, improved survival (Figure 6E, F), and significantly decreased viral titers and lung NP expression (Figure 6G, H).

Next, IAV strain H9N2 was utilized to infect the GAPLINC^+/-^ and WT mice. The data showed that, compared with WT controls, GAPLINC^+/-^ mice had significantly decreased viral NP mRNA and protein expression in lung tissues (Figure 6I, J). Given that mice are a well-established model for DNA virus PRV infection, the effect of GAPLINC deficiency on PRV gene expression was also examined. We found that significantly lower levels of the viral gE mRNA in GAPLINC^+/-^ mice compared to controls following infection with an equivalent dose of PRV (Figure 6K, L). Collectively, these observations suggest that heterozygous GAPLINC knockout mice display reduced susceptibility to IAV and PRV infection in vivo.

### Homozygous GAPLINC knockout mice exhibit increased resistance to IAV infection

**Figure 7:**
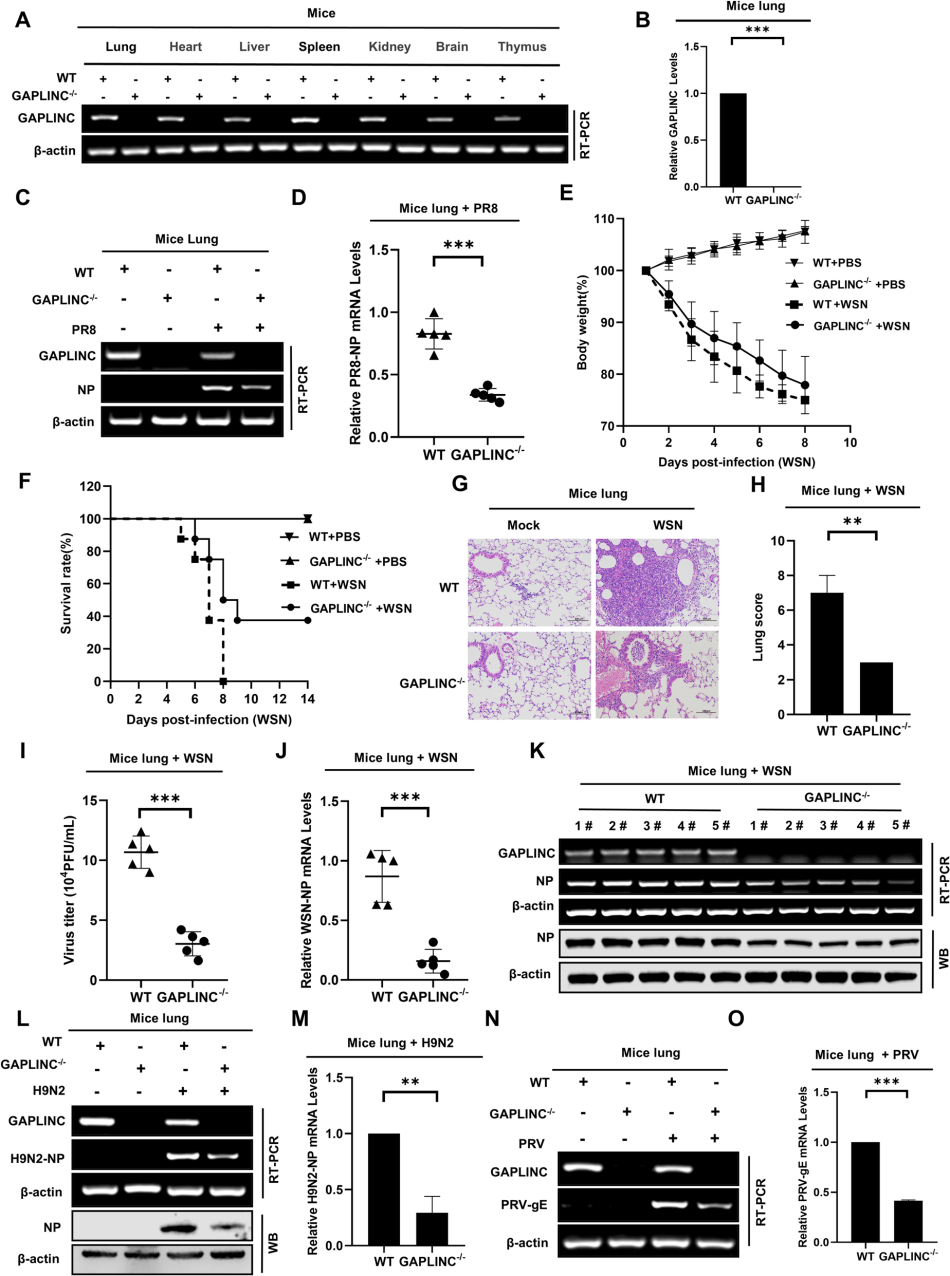
**Homozygous GAPLINC knockout mice exhibit increased resistance to IAV infection.** **A**, **B** GAPLINC RNA expression in the indicated tissues of WT and GAPLINC^⁻/⁻^ mice was detected by RT-PCR (**A**) and qRT-PCR (**B**, n = 3/group). **C**, **D** Analysis of viral NP mRNA in lungs of WT and GAPLINC^⁻/⁻^ mice at 48 hpi with PR8 (2 × 10^3^ PFU) by RT-PCR (**C**) and qRT-PCR (**D**, n = 5/group). **E**, **F** Body weight changes (**E**) and survival rates (**F**) of WT and GAPLINC^⁻/⁻^ mice (5–6 weeks, n = 8/group) infected with WSN (2 × 10^3^ PFU). **G**, **H** H&E-stained lung sections (**G)** and histopathology scores (**H**, n = 3/group) in WT and GAPLINC^⁻/⁻^ mice infected with WSN (2 × 10^3^ PFU) at 48 hpi. Bar = 200 µm. **I** Viral titers in lungs of WSN-infected (2 × 10^3^ PFU) WT and GAPLINC^⁻/⁻^ mice (n = 5/group) at 48 hpi measured by plaque assay. **J** NP mRNA levels in lung tissues assessed by qRT-PCR in WT and GAPLINC^⁻/⁻^ mice (n = 5/group) infected with WSN (2 × 10^3^ PFU) at 48 hpi. **K** NP mRNA and protein levels in lung tissues were assessed by RT-PCR and western blotting, respectively, in WT and GAPLINC^⁻/⁻^ mice (n = 5/group) infected with WSN (2 × 10^3^ PFU) at 48 hpi. **L**, **M** NP mRNA and protein levels in lung tissues were assessed by RT-PCR and western blotting in WT and GAPLINC^⁻/⁻^ mice infected with H9N2 (1 × 10^5^ PFU) at 48 hpi (**L**). Viral NP mRNA levels quantified by qRT-PCR (**M**, n = 3/group). **N**, **O** Viral PRV-gE mRNA levels from WT and GAPLINC^⁻/⁻^ mice infected with PRV (1 × 10^5^ PFU) at 48 hpi were detected by RT-PCR (**N**) and quantified by qRT-PCR (**O**, n = 3/group). Shown are representative data from three biologically independent experiments. Data are presented as means ± SD, **P < 0.01 and ***P < 0.001.

After further breeding, we obtained GAPLINC^⁻/⁻^ mice. Of note, the GAPLINC^⁻/⁻^ animals also exhibited no discernible phenotypic differences, including body weight, body length, coat condition, and locomotor activity, as compared to WT littermates (Additional file 5C). The RT-PCR analyses confirmed complete loss of GAPLINC expression in the heart, liver, spleen, lung, kidney, brain, and thymus of GAPLINC^⁻/⁻^ mice (Figure 7A, B; Additional file 5D). Initial infection with influenza virus PR8 revealed that GAPLINC^⁻/⁻^ mice had significantly lower viral NP expression in lung tissues than WT controls (Figure 7C, D). We then conducted a comprehensive analysis using the WSN strain. Following infection with IAV WSN, GAPLINC^⁻/⁻^ mice showed slower weight loss and higher survival rates than WT animals under the same conditions (Figure 7E, F). Consistently, significantly attenuated pulmonary tissue damage was observed in the GAPLINC^⁻/⁻^ mice compared to WT control challenged with the IAV infection (Figure 7G, H).

Next, we determined the effects of GAPLINC knockout on viral replication. The plaque assay showed that knockout of GAPLINC caused significantly reduced viral titers in lung tissues after WSN infection (Figure 7I). Likewise, markedly decreased viral NP mRNA and protein expressions were observed in GAPLINC^⁻/⁻^ mice compared to WT animals (Figure 7J, K). Moreover, two days post-influenza virus infection, GAPLINC^⁻/⁻^ mice exhibited reduced pulmonary hemorrhage and less severe splenic atrophy compared to WT mice (Additional file 5E, F). Subsequently, IAV strain H9N2 was used to verify these observations. Similarly, infection of mice with H9N2 revealed that GAPLINC^⁻/⁻^ mice had significantly lower viral NP expression in lung tissues than WT animals (Figure 7L, M). Additionally, the PRV infection model was employed and showed that viral gE expression was impaired in the GAPLINC^⁻/⁻^ mice compared with the control (Figure 7N, O). In addition, to directly compare the susceptibility of different mouse genotypes to IAV infection, WT, GAPLINC^+/-^, and GAPLINC^⁻/⁻^ mice were infected with the same dose of PR8, and lung tissues were collected at 48 hpi for qRT-PCR and western blotting analyses. Viral NP mRNA and protein levels showed a clear trend, with WT, GAPLINC^+/-^, and GAPLINC^⁻/⁻^ mice exhibiting the highest levels, intermediate levels, and the lowest levels of NP, respectively. These data support that GAPLINC deficiency is associated with progressively stronger suppression of PR8 infection in vivo (Additional file 5G, H). Collectively, these experiments demonstrate that genetic deletion of GAPLINC enhances murine resistance to IAV and PRV infection, leading to a significant alleviation of disease severity.

### Regulation of IAV infection by GAPLINC is associated with cellular autophagy

**Figure 8:**
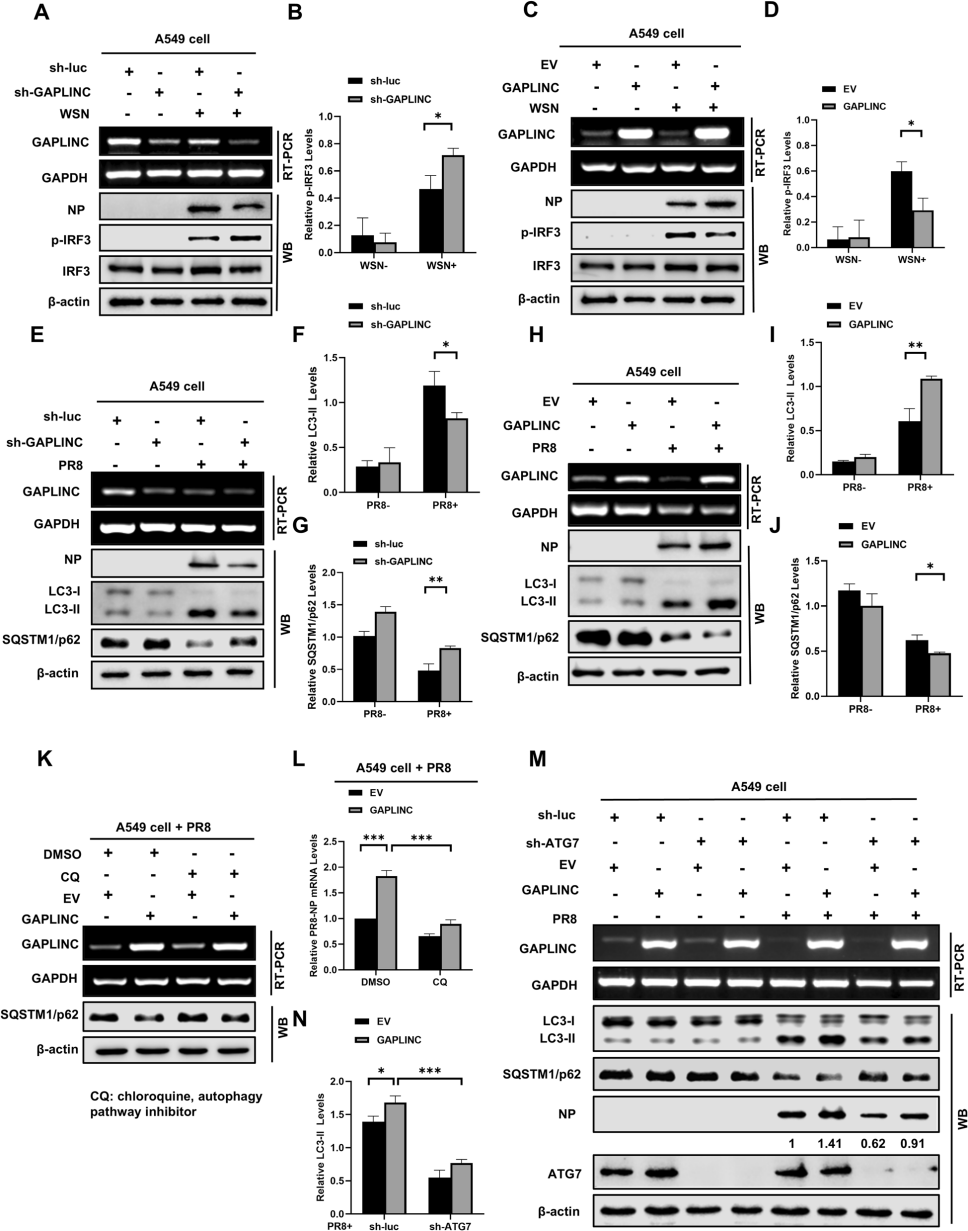
**Regulation of IAV infection by GAPLINC is associated with cellular autophagy.** **A**–**D** Analysis of GAPLINC knockdown (**A**, **B**) or GAPLINC overexpression (**C**, **D**) efficiency and autophagy markers in WSN (MOI = 1) infected A549 cells at 16 hpi by RT-PCR (**A**/**C**, upper panel). The levels of p-IRF3 in the cells were examined by western blotting (**A**/**C**, bottom panel) and semi-quantified using Image J (**B**/**D**, n = 3). **E**–**J** Analysis of GAPLINC knockdown (**E**–**G**) or GAPLINC overexpression (**H**–**J**) efficiency and autophagy markers in PR8 (MOI = 1) infected A549 cells at 16 hpi by RT-PCR (**E**/**H**, upper panel). The levels of NP, LC3 I/II, and SQSTM1/p62 in the cells examined by western blotting (**E**/**H**, bottom panel). LC3-II and SQSTM1/p62 bands semi-quantified using Image J (**F**/**G** and **I**/**J**, n = 3). **K**, **L** SQSTM1/p62 accumulation in GAPLINC-overexpressing A549 cells pretreated with 20 μM CQ for 2 h, followed by PR8 infection (MOI = 1) for 16 hpi in fresh maintenance medium. GAPLINC overexpression validated by RT-PCR; SQSTM1/p62 levels detected by western blotting (**K**). Viral replication levels quantified by qRT-PCR (**L**, n = 3). **M**, **N** Analysis of autophagy markers in ATG7-knockdown/GAPLINC-overexpressing A549 cells 16 h post-PR8 infection (MOI = 1). Validation by RT-PCR: ATG7 knockdown and GAPLINC overexpression; detection by western blotting: LC3-II/LC3-I ratio and SQSTM1/p62 levels (**M**). LC3-II band intensity semi-quantified using ImageJ (**N**, n = 3). Shown are representative data from three biologically independent experiments. Data are presented as means ± SD, *P < 0.05, **P < 0.01 and ***P < 0.001.

Our previous study has observed that GAPLINC negatively regulates the phosphorylation level of interferon regulatory factor 3 (IRF3) during IAV PR8 infection [29]. To validate this observation, we examined IRF3 phosphorylation in control and GAPLINC-knockdown A549 cells infected with another IAV strain, WSN. Indeed, the level of phosphorylated IRF3 was significantly higher in GAPLINC-knockdown cells than that in control cells after the viral infection, indicating that GAPLINC deficiency enhances IRF3 activation (Figure 8A, B). This finding was further verified in A549 cells overexpressing GAPLINC. As expected, overexpression of GAPLINC significantly suppressed IAV-induced IRF3 phosphorylation (Figure 8C, D).

As GAPLINC functions downstream of ATG7 [29], we further investigated whether GAPLINC regulates IAV infection involving the autophagy pathway. Knockdown of GAPLINC was found to suppress cellular autophagy, as indicated by reduced LC3-II and increased p62 levels (Figure 8E–G). In contrast, overexpression of GAPLINC significantly promoted autophagy (Figure 8H–J), indicating that GAPLINC may act as a positive regulator of autophagy. To define the relationship between autophagy and GAPLINC-mediated promotion of viral replication, cells overexpressing GAPLINC and control cells were pretreated with the autophagy inhibitor chloroquine (CQ) and subsequently infected with IAV PR8. The experiments demonstrated that CQ treatment abolished the enhancement of viral NP expression by GAPLINC overexpression compared to the control (Figure 8K, L), implying that the pro-viral function of GAPLINC is dependent on the execution of autophagy. Given that ATG7 positively regulates GAPLINC, we generated cell lines with concurrent ATG7 knockdown and GAPLINC overexpression. The results exhibited that GAPLINC overexpression alone promoted the autophagy and viral NP expression. However, in the context of ATG7 knockdown, such promotion effects were clearly attenuated (Figure 8M, N). Collectively, these findings suggest that GAPLINC facilitates IAV replication not only by suppressing IRF3 activation but also by promoting autophagy involving ATG7.

## Discussion

Although GAPLINC has previously been studied mainly in cancer and other pathological conditions, its role in viral infection is still poorly understood [37]. In the present study, we found that infection with diverse viruses induced a marked downregulation of GAPLINC expression in host cells, and that IAV infection similarly reduced GAPLINC expression in multiple organs of mice. The association between reduced GAPLINC expression and activation of the NF-κB/IL-6/STAT3 signaling axis suggests that inflammatory signaling contributes, at least in part, to the regulation of GAPLINC during infection. This interpretation is supported by our observations that IL-6 stimulation or overexpression of a constitutively active form of STAT3 reduced GAPLINC expression. Together, these findings place GAPLINC within a broader regulatory network linking cytokine signaling to host innate responses to viral infection. Nevertheless, whether additional signaling pathways also contribute to GAPLINC regulation in infected cells remains to be determined.

Functionally, our data support a proviral role of GAPLINC in the context of IAV infection. In cultured cells, increased GAPLINC expression was associated with enhanced viral replication and cytopathic effects, whereas reduced GAPLINC expression suppressed these effects. Similarly, in vivo studies showed that decreased GAPLINC expression was associated with reduced viral burden and attenuated disease manifestations after IAV infection, supporting the notion that GAPLINC contributes to viral replication and pathogenesis. Our results further suggest that GAPLINC facilitates IAV infection at a post-entry stage and that its proviral effect is mediated, at least in part, through suppression of IRF3 activation. Collectively, these findings indicate that GAPLINC may function as a positive regulator of IAV infection through modulation of host innate antiviral responses.

Autophagy is increasingly recognized as an important host process during viral infection and has been reported to exert complex and context-dependent effects on both viral replication and antiviral immunity [25, 38–40]. On the one hand, autophagy may serve as a cellular defense mechanism by promoting degradation of viral components, regulating inflammatory signaling, and maintaining cellular homeostasis under infection-induced stress. On the other hand, viruses may also exploit autophagy-related machinery to support replication or persistence, indicating that the role of autophagy in viral infection is neither purely antiviral nor purely proviral, but instead depends on the stage of infection, host cell type, and signaling context. In this study, our data suggest that GAPLINC promotes viral replication, at least in part, through positive regulation of the autophagy pathway. This places GAPLINC at the intersection of innate antiviral signaling and autophagy regulation during IAV infection. Our findings therefore raise the possibility that GAPLINC facilitates IAV replication through coordinated modulation of host antiviral responses and autophagic activity. Nevertheless, the precise molecular mechanisms by which GAPLINC suppresses IAV-triggered IRF3 phosphorylation and promotes cellular autophagy remain to be elucidated.

Given its proviral role, the observed downregulation of GAPLINC during virus-host interaction may reflect a complex bidirectional regulatory process. On the one hand, reduced GAPLINC expression may represent a host-driven defense mechanism that helps restrict viral infection by permitting more rapid activation of antiviral responses. On the other hand, the dynamic regulation of GAPLINC during infection may also reflect a more complex adaptation at the host–pathogen interface. Thus, our findings suggest that GAPLINC may participate in the fine-tuning of antiviral immunity during infection.

In addition to IAV, we observed that GAPLINC expression was also downregulated following infection with other viruses, including the RNA virus SeV and the DNA viruses PRV and HSV. Previous studies have shown that PRV and HSV can interact with autophagy-related pathways during infection [41–43]. These observations raise the possibility that the downregulation of GAPLINC is not restricted to IAV infection, but may instead reflect a broader virus-associated host response. Given that our data support a role for GAPLINC in promoting IAV replication partly through autophagy, it is possible that GAPLINC may also participate in the infection of these other viruses through related mechanisms. Although a broad panel of viruses was included in the initial screening, subsequent functional analyses were concentrated on representative viruses, including PR8, WSN, H3N2, H9N2, and PRV. However, the effects of GAPLINC knockdown on other tested viruses, such as SeV and HSV, were not systematically evaluated in the present study. Therefore, it remains unclear whether the regulatory role of GAPLINC is broadly conserved or varies across different viral infection models. Further investigation will be required to determine whether GAPLINC exerts a similar regulatory role in these additional viral infection models.

Although IAV infect a wide range of host species, including birds and mammals, the present study was performed in human cells and mouse models, and therefore our findings mainly support a role for GAPLINC in mammalian influenza infection models. In this regard, GAPLINC has been reported to be functionally conserved between human and mouse [32], whereas its presence and functional relevance in avian species remain unclear. Moreover, several of the influenza viruses used in this study, such as PR8 and WSN, are classical laboratory strains widely used in mammalian infection models, whereas H3N2 is a human seasonal influenza subtype and H9N2 is an avian-origin subtype with known zoonotic and mammalian adaptation potential [44]. Accordingly, the GAPLINC-influenza connection described here should be interpreted primarily within the context of the mammalian systems examined in this study rather than generalized to all influenza host species.

Interestingly, the present findings may also have a broader conceptual connection to the previously reported role of GAPLINC in cancer [37]. GAPLINC has been implicated in tumor-associated processes such as inflammatory signaling, transcriptional regulation, and cellular adaptation to pathological stress. In our study, GAPLINC was associated with regulation of NF-κB/IL-6/STAT3 signaling, IRF3 activation, and autophagy, suggesting that GAPLINC may function more generally as a regulator of host cellular states that can be exploited in distinct pathological contexts. Although the relationship between the protumor and proviral functions of GAPLINC remains unclear, our data raise the possibility that this lncRNA contributes to disease progression by shaping intracellular environments that favor either tumor development or viral replication. Further studies will be needed to determine whether these apparently distinct functions of GAPLINC share common molecular mechanisms.

In summary, this study identifies lncRNA GAPLINC as an important host factor that enhances the infection and pathogenesis of IAV in the human and mouse systems examined here. We show that GAPLINC expression is downregulated by IAV infection in human cells and mouse tissues, and that this suppression is associated with activation of the NF-κB/IL-6/STAT3 signaling axis. Functionally, GAPLINC acts as a proviral factor and promotes viral replication at a post-entry stage, at least in part through suppression of IRF3 activation and enhancement of autophagy. Overall, our work identifies GAPLINC as a host regulatory lncRNA involved in influenza virus infection and suggests that its virus-induced downregulation may represent a host-associated protective response, highlighting GAPLINC as a potential target for antiviral intervention.

## Supplementary Information

**Additional file 1: Comparative analysis of the genomic conservation and annotated transcripts of the GAPLINC gene**. (A) Both human GAPLINC and murine GAPLINC are located between Tgif1 and Dlgap1, demonstrating conserved genomic synteny. (B) Two splice variants of the GAPLINC transcript are currently annotated in the NCBI database. C Fourteen transcript variants of GAPLINC are currently annotated in the Ensembl database.

**Additional file 2: Analysis of GAPLINC expression upon viral challenge.** (A, B) PR8 infection in A549 cells at indicated MOIs (0, 0.3, 1, 3) harvested at 16 hpi (A) or at MOI = 1 harvested at 0, 8, 16, and 24 hpi (B). NP mRNA levels were measured by qRT-PCR (n = 3). (C, E, G) qRT-PCR analysis of viral NP mRNA levels in A549 cells at 16 hpi with WSN (MOI = 1) (C, n = 3), H3N2 (MOI = 1) (E, n = 3), or H9N2 (MOI = 1) (G, n = 3). (D, F, H) GAPLINC expression in A549 cells infected with WSN (D), H3N2 (F), or H9N2 (H) at 16 hpi, analyzed by RT-PCR. (I-J) NP mRNA levels in HEK293T (I) and HeLa (J) cells infected with WSN at MOI = 1 for 16 h (qRT-PCR, n = 3). (K, M, O) qRT-PCR analysis of SeV-NP, PRV-gE, and HSV-gC mRNA levels in A549 cells at 16 hpi with SeV (MOI = 1) (M, n = 3), PRV (MOI = 1) (O, n = 3), or HSV (MOI = 1) (Q, n = 3). (L, N) GAPLINC expression in A549 cells infected with SeV (L) or PRV (N) at 16 hpi, analyzed by RT-PCR. (P) qRT-PCR analysis of viral NP mRNA levels in the indicated tissues of WT mice (5-6 weeks) at 48 h post intranasal infection with PR8 (2 × 10^3^ PFU, n = 3). (Q) RT-PCR analysis of GAPLINC levels in lung tissues of WT mice (5-6 weeks) following intranasal infection with PR8 (2 × 10^3^ PFU). (R) Representative RT-PCR images showing GAPLINC and viral NP levels in the indicated tissues of WT mice  (5-6 weeks), either non-infected (mock) or infection with PR8 for 48 h. Shown are representative data from three biologically independent experiments. Data are presented as means ± SD, *P < 0.05 and ***P < 0.001.

**Additional file 3: GAPLINC modulates cell death and viral NP expression upon PR8 infection.** (A) Representative phase-contrast micrographs of control and GAPLINC-knockdown A549 cells, either mock-infected or infected with PR8 virus (MOI = 1) at 36 hpi. Bar = 200 µm. (B) Representative fluorescence images of control and GAPLINC-knockdown A549 cells infected with PR8 virus (MOI = 1) at 36 hpi, as stained with propidium iodide (PI). PI-positive (red) signals indicate dead cells. Bar = 200 µm. (C) The percentage of PI-positive cells from multiple random fields (B) was quantified (n = 3). (D) Viral NP mRNA levels in PR8 (MOI = 1) infected A549 cells with GAPLINC-knockdown or control at the indicated time points (2, 4, 6 hpi, n = 3). (E) Representative phase-contrast micrographs of control and GAPLINC-overexpressing A549 cells infected with or without with PR8 virus (MOI = 1) at 36 hpi. Bar = 200 µm. (F) Representative fluorescence images of control and GAPLINC-overexpressing A549 cells infected with the influenza PR8 virus (MOI = 1) at 36 hpi, as stained with PI. PI-positive (red) signals indicate dead cells. Bar = 200 µm. (G) The percentage of PI-positive cells (F) from multiple random fields was quantified (n = 3). Shown are representative data from three biologically independent experiments. Data are presented as means ± SD, not significant (ns) and ***P < 0.001.

**Additional file 4: Effects of GAPLINC knockdown or overexpression on PR8 influenza virus attachment, internalization, and RNA synthesis.** (A) GAPLINC-knockdown and control A549 cells were incubated with PR8 virus (MOI = 10) at 4 °C for 1 h to allow synchronous viral attachment while preventing internalization. The levels of cell-associated vRNA for the viral NP and M genes were quantified by qRT-PCR (n = 3). (B) GAPLINC-overexpressing and control A549 cells were incubated with PR8 virus (MOI = 10) at 4 °C for 1 h to allow synchronous viral attachment while preventing internalization. The levels of cell-associated vRNA for the viral NP and M genes were quantified by qRT-PCR (n = 3). (C) GAPLINC-knockdown and control A549 cells were incubated with PR8 virus (MOI = 10) attachment at 4 °C for 1 h, followed by a temperature shift to 37 °C for 30 min to allow internalization. The levels of internalized vRNA for the NP and M genes were then quantified by qRT-PCR (n = 3). (D) GAPLINC-overexpressing and control A549 cells were incubated with PR8 virus (MOI = 10) attachment at 4 °C for 1 h, followed by a temperature shift to 37 °C for 30 min to allow internalization. The levels of internalized vRNA for the NP and M genes were then quantified by qRT-PCR (n = 3). (E-G) qRT-PCR analysis of vRNA (E), cRNA (F), and mRNA (G) levels for both NP and M genes in control and GAPLINC-knockdown A549 cells infected with PR8 at 16 hpi (MOI = 1, n = 3). Shown are representative data from three biologically independent experiments. Data are presented as means ± SD, not significant (ns), **P < 0.01 and ***P < 0.001.

**Additional file 5: Generation and influenza virus infection of GAPLINC knockout mice.** (A) Schematic of the genomic editing site in GAPLINC knockout mice. The blue arrow indicates the deleted region. (B) Comparison of phenotypic characteristics between GAPLINC^+/-^ mice and WT mice. (C) Comparison of phenotypic characteristics between GAPLINC^⁻/⁻^ mice and WT mice. (D) RT-PCR analysis of GAPLINC RNA expression in lung tissues from WT and GAPLINC^⁻/⁻^ mice. (E,F) Lungs and spleens harvested from WT and GAPLINC^⁻/⁻^ mice at 48 h post intranasal inoculation with WSN virus or PBS (control). Representative images from three independent experiments are shown. (G) NP mRNA levels in lung tissues assessed by qRT-PCR in WT, GAPLINC^+/-^, and GAPLINC^⁻/⁻^ mice (n = 5/group) infected with PR8 (2 × 10^3^ PFU) at 48 hpi. (H) NP mRNA and protein levels in lung tissues were assessed by RT-PCR and western blotting, respectively, in WT, GAPLINC^+/-^, and GAPLINC^⁻/⁻^ mice (n = 5/group) infected with PR8 (2 × 10^3^ PFU) at 48 hpi. Shown are representative data from three biologically independent experiments. Data are presented as means ± SD, **P < 0.01 and ***P < 0.001.

## Data Availability

All the data generated or analyzed in this study are available in this published article and its supplementary information files.
