# Supplementary figures and images for: In vitro and in vivo studies of GAPLINC identify it as a critical host factor involved in the regulation of influenza A virus infection
Source: Vet Res. 2026 Jul 16;57:136. doi: 10.1186/s13567-026-01783-1 (PMC13377811; doi:10.1186/s13567-026-01783-1)

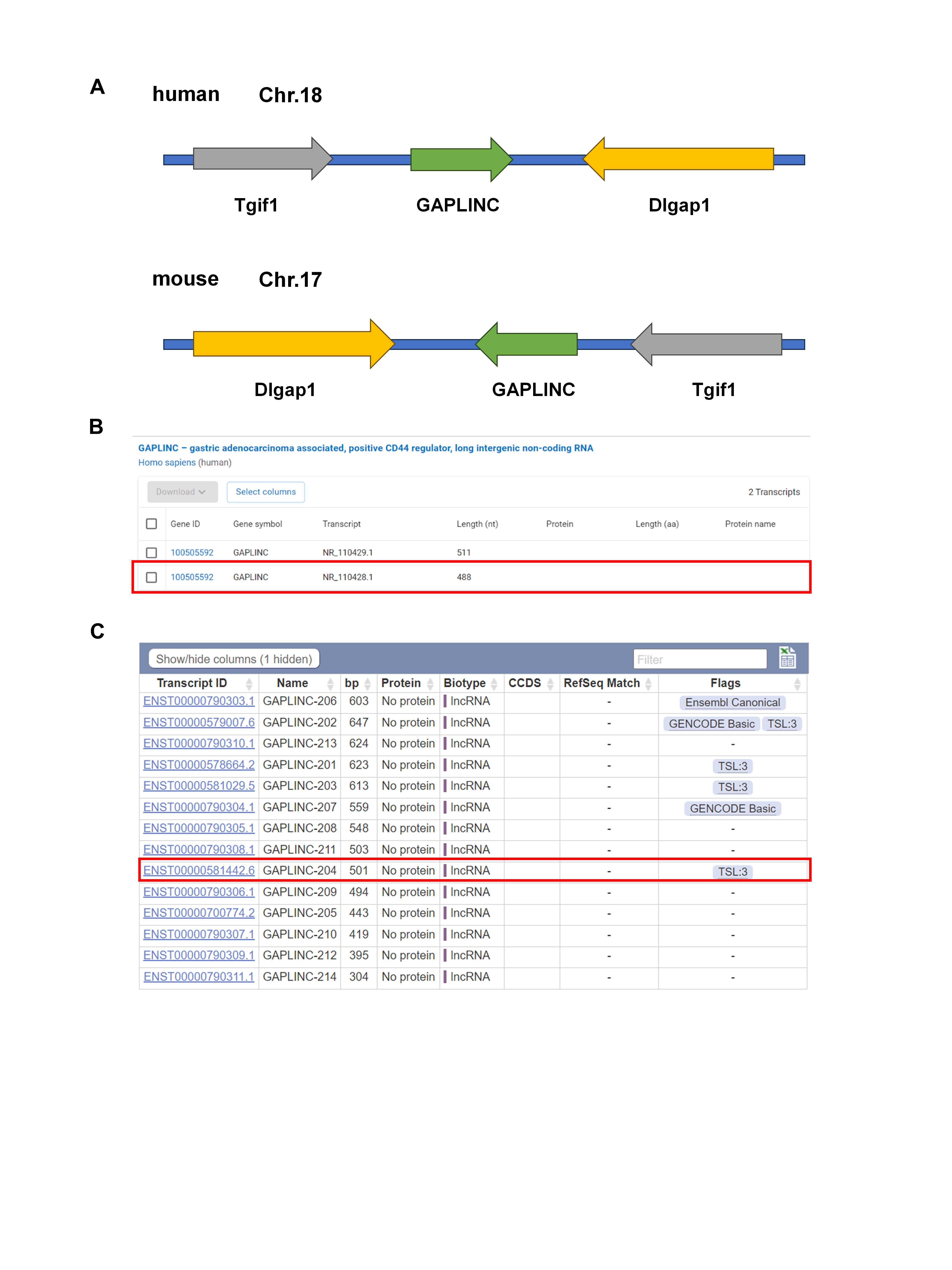

Supplement: Supplementary file 1 — Additional file 1: Comparative analysis of the genomic conservation and annotated transcripts of the GAPLINC gene. (A) Both human GAPLINC and murine GAPLINC are located between Tgif1 and Dlgap1, demonstrating conserved genomic synteny. (B) Two splice variants of the GAPLINC transcript are currently annotated in the NCBI database. C Fourteen transcript variants of GAPLINC are currently annotated in the Ensembl database. [file 13567_2026_1783_MOESM1_ESM.tif]

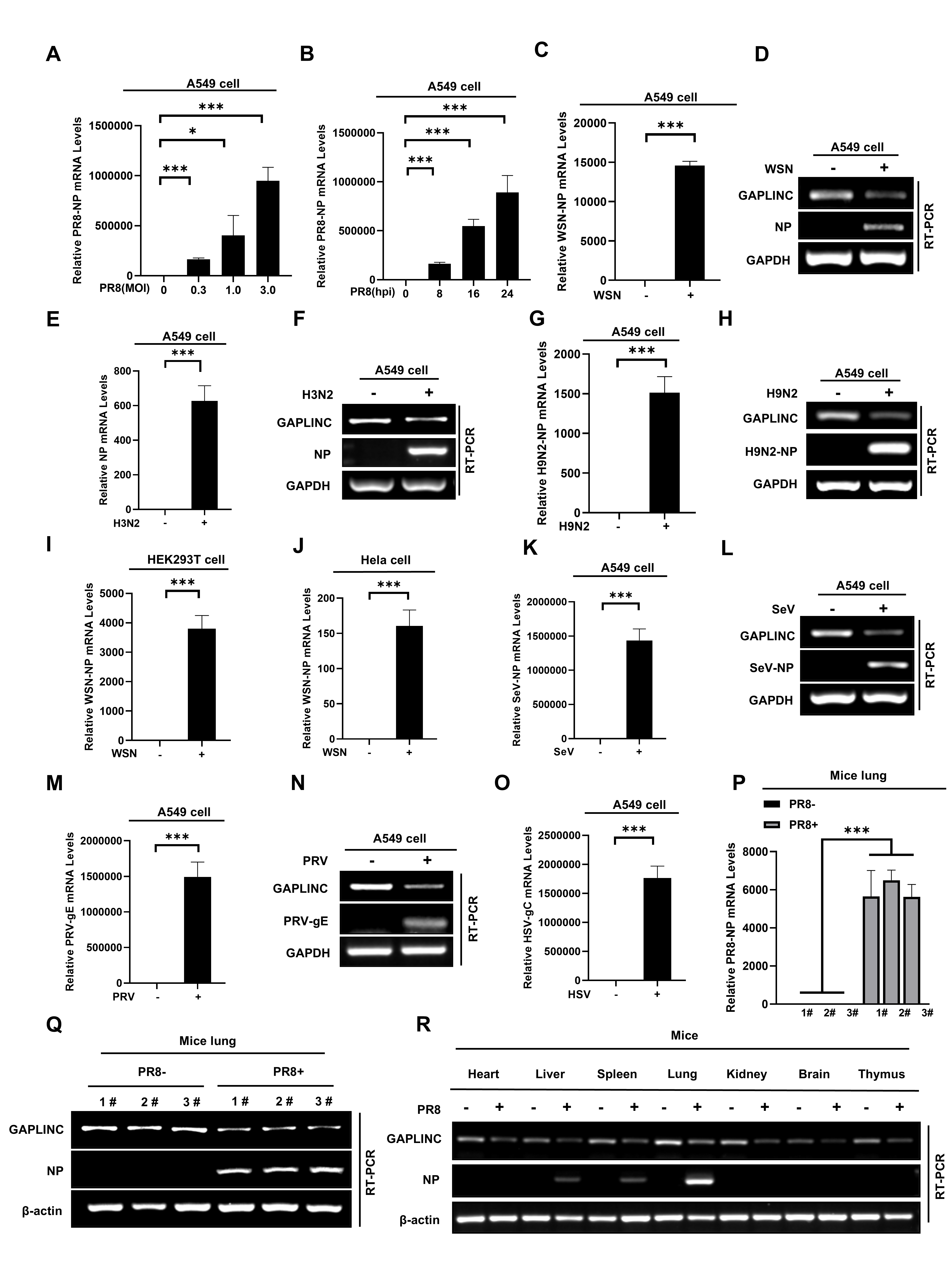

Supplement: Supplementary file 2 — Additional file 2: Analysis of GAPLINC expression upon viral challenge. (A, B) PR8 infection in A549 cells at indicated MOIs (0, 0.3, 1, 3) harvested at 16 hpi (A) or at MOI = 1 harvested at 0, 8, 16, and 24 hpi (B). NP mRNA levels were measured by qRT-PCR (n = 3). (C, E, G) qRT-PCR analysis of viral NP mRNA levels in A549 cells at 16 hpi with WSN (MOI = 1) (C, n = 3), H3N2 (MOI = 1) (E, n = 3), or H9N2 (MOI = 1) (G, n = 3). (D, F, H) GAPLINC expression in A549 cells infected with WSN (D), H3N2 (F), or H9N2 (H) at 16 hpi, analyzed by RT-PCR. (I-J) NP mRNA levels in HEK293T (I) and HeLa (J) cells infected with WSN at MOI = 1 for 16 h (qRT-PCR, n = 3). (K, M, O) qRT-PCR analysis of SeV-NP, PRV-gE, and HSV-gC mRNA levels in A549 cells at 16 hpi with SeV (MOI = 1) (M, n = 3), PRV (MOI = 1) (O, n = 3), or HSV (MOI = 1) (Q, n = 3). (L, N) GAPLINC expression in A549 cells infected with SeV (L) or PRV (N) at 16 hpi, analyzed by RT-PCR. (P) qRT-PCR analysis of viral NP mRNA levels in the indicated tissues of WT mice (5-6 weeks) at 48 h post intranasal infection with PR8 (2 × 10^3^ PFU, n = 3). (Q) RT-PCR analysis of GAPLINC levels in lung tissues of WT mice (5-6 weeks) following intranasal infection with PR8 (2 × 10^3^ PFU). (R) Representative RT-PCR images showing GAPLINC and viral NP levels in the indicated tissues of WT mice (5-6 weeks), either non-infected (mock) or infection with PR8 for 48 h. Shown are representative data from three biologically independent experiments. Data are presented as means ± SD, *P < 0.05 and ***P < 0.001. [file 13567_2026_1783_MOESM2_ESM.tif]

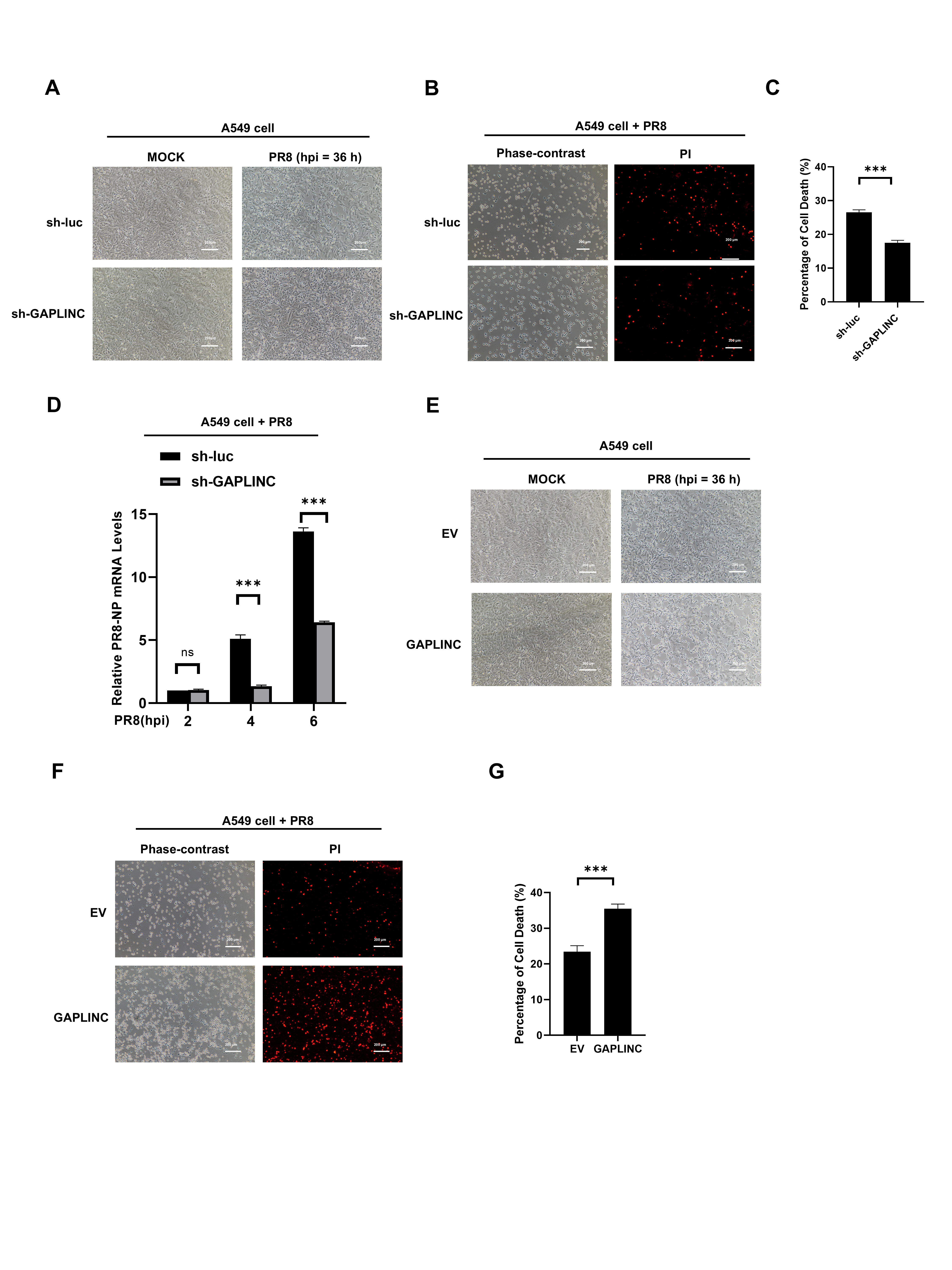

Supplement: Supplementary file 3 — Additional file 3: GAPLINC modulates cell death and viral NP expression upon PR8 infection. (A) Representative phase-contrast micrographs of control and GAPLINC-knockdown A549 cells, either mock-infected or infected with PR8 virus (MOI = 1) at 36 hpi. Bar = 200 µm. (B) Representative fluorescence images of control and GAPLINC-knockdown A549 cells infected with PR8 virus (MOI = 1) at 36 hpi, as stained with propidium iodide (PI). PI-positive (red) signals indicate dead cells. Bar = 200 µm. (C) The percentage of PI-positive cells from multiple random fields (B) was quantified (n = 3). (D) Viral NP mRNA levels in PR8 (MOI = 1) infected A549 cells with GAPLINC-knockdown or control at the indicated time points (2, 4, 6 hpi, n = 3). (E) Representative phase-contrast micrographs of control and GAPLINC-overexpressing A549 cells infected with or without with PR8 virus (MOI = 1) at 36 hpi. Bar = 200 µm. (F) Representative fluorescence images of control and GAPLINC-overexpressing A549 cells infected with the influenza PR8 virus (MOI = 1) at 36 hpi, as stained with PI. PI-positive (red) signals indicate dead cells. Bar = 200 µm. (G) The percentage of PI-positive cells (F) from multiple random fields was quantified (n = 3). Shown are representative data from three biologically independent experiments. Data are presented as means ± SD, not significant (ns) and ***P < 0.001. [file 13567_2026_1783_MOESM3_ESM.tif]

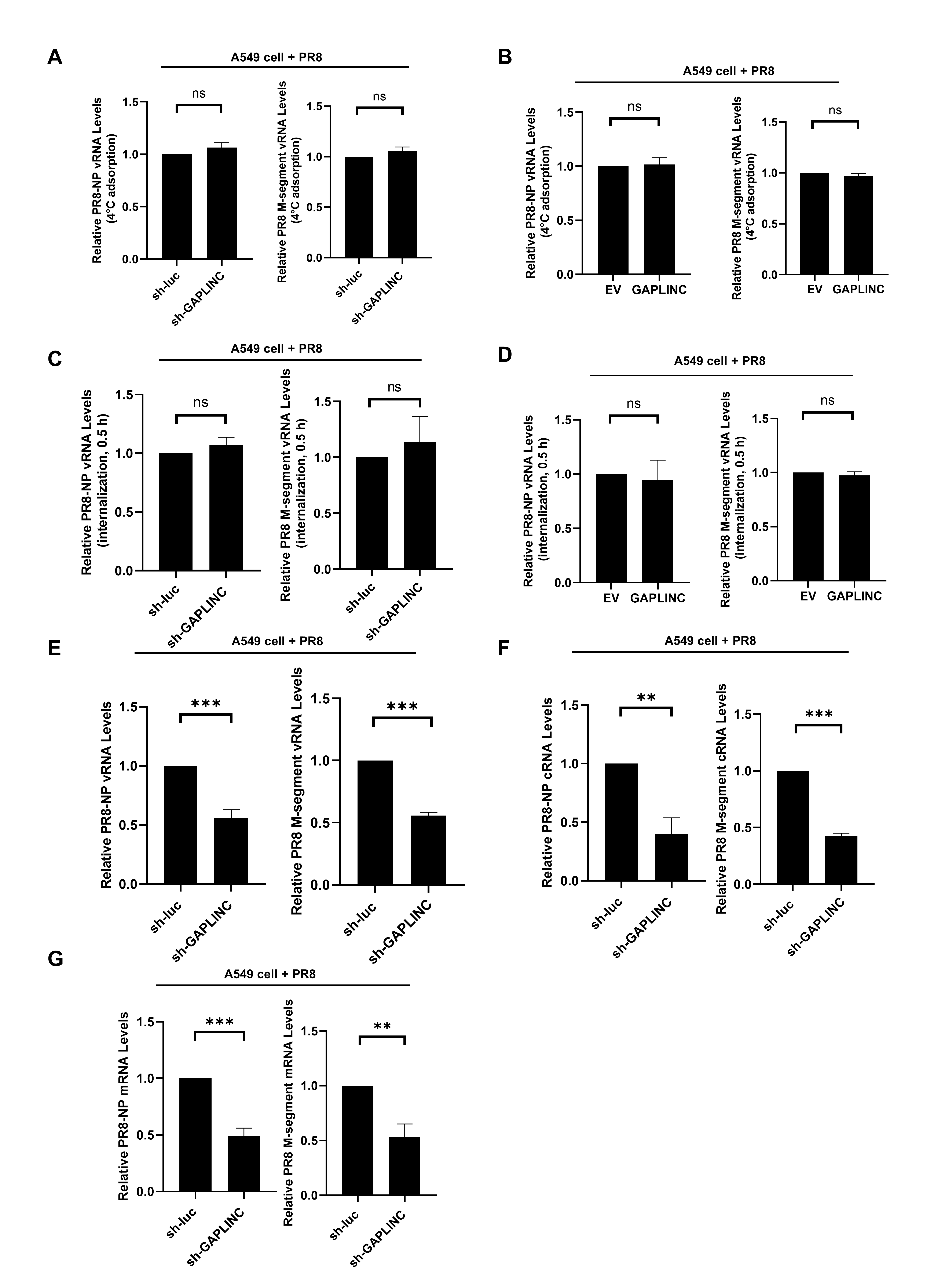

Supplement: Supplementary file 4 — Additional file 4: Effects of GAPLINC knockdown or overexpression on PR8 influenza virus attachment, internalization, and RNA synthesis. (A) GAPLINC-knockdown and control A549 cells were incubated with PR8 virus (MOI = 10) at 4 °C for 1 h to allow synchronous viral attachment while preventing internalization. The levels of cell-associated vRNA for the viral NP and M genes were quantified by qRT-PCR (n = 3). (B) GAPLINC-overexpressing and control A549 cells were incubated with PR8 virus (MOI = 10) at 4 °C for 1 h to allow synchronous viral attachment while preventing internalization. The levels of cell-associated vRNA for the viral NP and M genes were quantified by qRT-PCR (n = 3). (C) GAPLINC-knockdown and control A549 cells were incubated with PR8 virus (MOI = 10) attachment at 4 °C for 1 h, followed by a temperature shift to 37 °C for 30 min to allow internalization. The levels of internalized vRNA for the NP and M genes were then quantified by qRT-PCR (n = 3). (D) GAPLINC-overexpressing and control A549 cells were incubated with PR8 virus (MOI = 10) attachment at 4 °C for 1 h, followed by a temperature shift to 37 °C for 30 min to allow internalization. The levels of internalized vRNA for the NP and M genes were then quantified by qRT-PCR (n = 3). (E-G) qRT-PCR analysis of vRNA (E), cRNA (F), and mRNA (G) levels for both NP and M genes in control and GAPLINC-knockdown A549 cells infected with PR8 at 16 hpi (MOI = 1, n = 3). Shown are representative data from three biologically independent experiments. Data are presented as means ± SD, not significant (ns), **P < 0.01 and ***P < 0.001. [file 13567_2026_1783_MOESM4_ESM.tif]

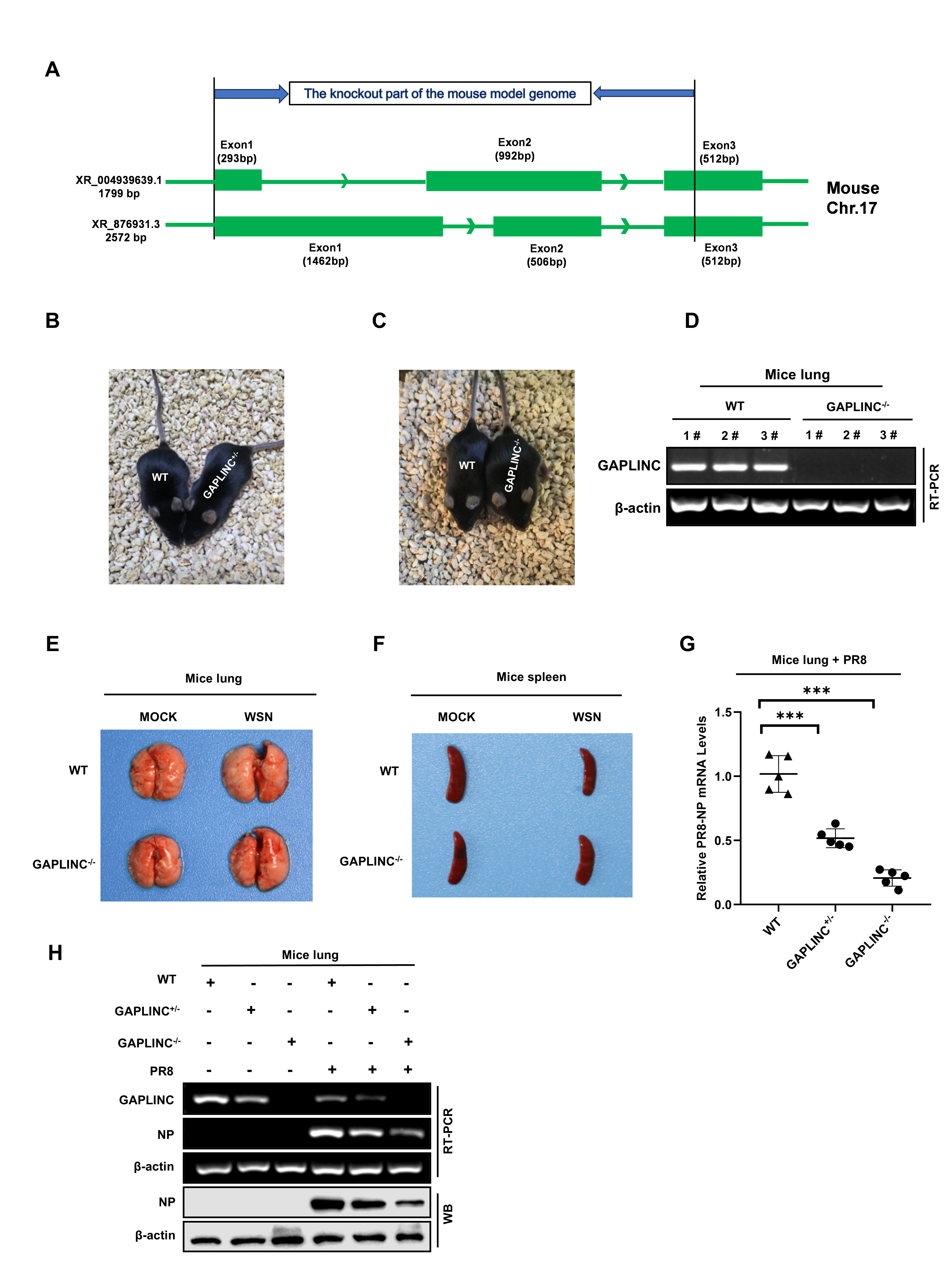

Supplement: Supplementary file 5 — Additional file 5: Generation and influenza virus infection of GAPLINC knockout mice. (A) Schematic of the genomic editing site in GAPLINC knockout mice. The blue arrow indicates the deleted region. (B) Comparison of phenotypic characteristics between GAPLINC+/- mice and WT mice. (C) Comparison of phenotypic characteristics between GAPLINC⁻/⁻ mice and WT mice. (D) RT-PCR analysis of GAPLINC RNA expression in lung tissues from WT and GAPLINC⁻/⁻ mice. (E,F) Lungs and spleens harvested from WT and GAPLINC⁻/⁻ mice at 48 h post intranasal inoculation with WSN virus or PBS (control). Representative images from three independent experiments are shown. (G) NP mRNA levels in lung tissues assessed by qRT-PCR in WT, GAPLINC+/-, and GAPLINC⁻/⁻ mice (n = 5/group) infected with PR8 (2 × 10^3^ PFU) at 48 hpi. (H) NP mRNA and protein levels in lung tissues were assessed by RT-PCR and western blotting, respectively, in WT, GAPLINC+/-, and GAPLINC⁻/⁻ mice (n = 5/group) infected with PR8 (2 × 10^3^ PFU) at 48 hpi. Shown are representative data from three biologically independent experiments. Data are presented as means ± SD, **P < 0.01 and ***P < 0.001. [file 13567_2026_1783_MOESM5_ESM.tif]
